# Supplementary material for: Allosteric regulation and crystallographic fragment screening of SARS-CoV-2 NSP15 endoribonuclease
Source: Nucleic Acids Res. 2023 Apr 28;51(10):5255–70. doi: 10.1093/nar/gkad314 (PMC10250223; doi:10.1093/nar/gkad314)
Supplement: gkad314_Supplemental_Files [file gkad314_supplemental_files.zip › Supplementary datasets.zip!/217/Refine_9/01-BUSTER/Cycle-2/LIST.html]

Refining

**BUSTER 2.X**: A suite of programs for macromolecular structure refinement by maximum-likelihood and Bayesian methods  
Copyright © 1992-2020 Global Phasing Ltd.  
All rights reserved.  
Authors: G. Bricogne, E. Blanc, M. Brandl, C. Flensburg, P. Keller, W. Paciorek, P. Roversi, O. Smart, C. Vonrhein, T. Womack  

Please reference use of the software as:

- BUSTER-TNT 2.X, Global Phasing Ltd, Sheraton House, Cambridge CB3 0AX, UK

Version: 2.10.3 for linux64-ifort made on May 20 2020 at 01:14:34  
Licenced to: Diamond Light Source, Scientific Software  

```
---


### Parsing of the BUSTER cardfile

TITLE : autoBUSTER refinement

   Cell information

   Symmetry information

 Using CCP4 X-ray form factors.

   Missing structure chemical composition

 Using ATOM block for chemical composition.


   Shell information

### Parsing of the TNT cardfile


---


### Reading observations file

 Number of reflexions in input:  47009

      Number of accepted reflexions:  42835

      Number of rejected reflexions:      0

   Reflexions in test set:            2171

   Reflexions in working set:        40664


---


### 1. Generating initial model

 2.10 * Shannon sampling rate. NX NY NZ:   288  288  192


  Effective Shannon rates are:    2.44    2.44    2.23


   Allocated BUFFER of size:     61.172 Mb.

   Initial Geometry Information


 No missing atoms declared


   Summary of model volumes

 No missing atoms declared: no prior computed


 Computing bulk solvent model scattering


 NCSMASK PDB radius for solvent mask:     2.15 Angstrom

 running PDBSET (setting cell and spacegroup P1) ... done
 running NCSMASK [1] (creating mask, SMOOTH 1.14715) ... done
 running MAPDUMP (to check if reduction to unit cell is needed) ... done
 running MAPMASK (converting mask to map) ... done

   Generation of babslv by theta-filter

   Electron density in frg mask:  0.420 Å-1

 FOBS  put on rough absolute scale by Wilson plot :
           K        =  0.71102E+02
           B        =     -5.418

 

### Cycle 0001: MaxLik scaling and refinement


   MaxLik Scaling Cycle 0001

   Wilson plot

   Statistics in resolution bins just after scaling
```

|  |  |
| --- | --- |
|  | - Rxpct factors plot- Rxpct factors table- <Log-Lik Gain>- Ln Sigma A- Correlation Coefficients |

```
   <Log-Likelihood> of null-hypothesis

Structure Factor Amplitudes


   Average Figure of Merit

 Switching off refinement of overall temperature factor


 Added B =     0.1635 Å2 to all atoms in fragment


 Added B =     0.1635 Å2 to B_SOLV

```
gelly refinement module
 Authors (2004-2009) O. S. Smart, C. Flensburg, W. Paciorek, C. Vonrhein, 
                     M. Brandl, T. O. Womack and G. Bricogne
         (2010-2011) O. S. Smart, C. Flensburg, W. Paciorek, C. Vonrhein, 
                     T. O. Womack and G. Bricogne
         (2012-2014) O. S. Smart, C. Flensburg, W. Paciorek, C. Vonrhein, 
                     and G. Bricogne
         (2015-2020) G. Bricogne, C. Flensburg, W. Paciorek, C. Vonrhein
 Copyright © 2004-2020 Global Phasing Ltd. 
 All rights reserved.
 gelly module compilation date May 20 2020 01:11:20
 
 gelly uses routines from TNT geometry. References: 
     Tronrud, D. E., Ten Eyck, L. F., Matthews, B. W. (1987)
     "An Efficient General-Purpose Least-Squares Refinement Program 
     for Macromolecular Structures". Acta Crystallogr A, 43:489-501.
     Tronrud, D. E. (1997). "The TNT Refinement Package", 
     in Macromolecular Crystallography, Part B, Eds Charlie Carter 
     and Robert Sweet, Methods in Enzymology, 277:306-319.
 wildcard matching function by Julian Robichaux http://www.nsftools.com

 No -random_seed flag found. Random number stream initialised using:
2147483562
2147483398

 Control flags set to:  -autoncs -hydrogens_input_occ2zero \
                     -hydrogens_output_occ2heavy -screen \
                     100 -screen_sigma \
                     3.0 -keeppdb \
                     1000000 -taomonitor_verbose \
                     2 
 

 ############### Starting setup via TNT routines

 Setting up tnt routines including license check:

                   TNT Stereochemistry Program
 Version: 5.22.3.0
 Licence for Diamond Light Source, Scientific Software is valid
 
 Using TNT geometry routines to load the tnt control file: gelly.tnt
 Using TNT routines to load initial coordinates from file fragment.cor
 Expecting 6517 atoms

 Found     2191 "NOTE BUSTER_RESIDUE_ATOM_DATA" cards
 Have copied information for    6517 atoms from TNT to gelly's store 
 
 The TNT WEIGHT cards used by this run:
 -----WEIGHT       BOND     2.0
 -----WEIGHT       ANGLE    2.0
 -----WEIGHT       IMPROPER 0.0
 -----WEIGHT       TORSION  2.0
 -----WEIGHT       PSEUDO   0.0
 -----WEIGHT       TRIGONAL 2.0
 -----WEIGHT       PLANE    5.0
 -----WEIGHT       CONTACT  5.0
 -----WEIGHT       BCORREL  10.0
 -----WEIGHT       CHIRAL   5.0
 -----WEIGHT       NCS      50.0
 -----WEIGHT       IDEAL    4.0
 -----WEIGHT       RFACTOR  9.39
 
 Dictionary versions picked up from NOTE BUSTER_RESTRAINT_DICT cards as: 
 ------ protgeo_eh99.dat (V1.6.4.17) 20191001  STANDARD AMINO ACID DICTIONARY. BONDS AND ANGLES FROM ENGH AND HUBER EH99. OTHER VALUES BASED ON PREVIOUS TNT OR TAKEN FROM CCP4. INCLUDES HYDROGEN ATOMS.
 ------ exoticaa.dat (V1.3.4.15) 20200327  COLLECTION OF NON-STANDARD AMINO ACIDS, MAINLY EH91 WITHOUT IDEAL DISTANCE INFO
 ------ nuclgeo.dat (V1.13.4.8) 20190909
 ------ bcorrel.dat (V1.15.10.2) 20190616
 ------ contact.dat (V1.15.12.10) 20191121
 ------ idealdist_contact.dat (V1.3.4.12) 20191001  IDEAL-DISTANCE CONTACT TERM DATA AS USED IN PROLSQ. VALUES USED HERE ARE BASED ON DATA HARVESTING OF PDB AS OF 20190109.
 ------ restraints for LIG (Unknown) from cif dictionary Z1269638430.cif
 ------ restraints for CIT (CITRIC ACID) from cif dictionary CIT.cif; generated by GRADE_PDB_LIGAND 1.2.12 (October 4 2016) using MOGUL 1.7.2(RC1), CSD as538be, with quantum mechanics RM1 ((DatabaseID PDB-CIT))
 ------ assume.dat (V1.9.4.2) 20180705
 
 Have picked up spacegroup name for pdb output as 'P 63      '
 Setting up Chain/Residue arrays
 
 Have command line specification -autoncs*
     Have found       1 NCS relations
     Pairwise matching residue number and type between chains: 
                    A        B        S        W    
          A         .      100%       -        -   
          B                  .        -        -   
          S                           .        -   
          W                                    .   
       Table key--                                                       
          all   : two chains have 100% match and same number of residues 
         multi  : ditto and they contribute to a multi chain ncs cluster 
          ***%  : two chains have >80% matching residue number/type      
           -    : chains appear to be unrelated                          
      chain A     have identified ncs to     1 other chains. So will weight each autoncs by (       6.666/#)=      6.6660
      chain B     have identified ncs to     1 other chains. So will weight each autoncs by (       6.666/#)=      6.6660
      chain S     have identified ncs to     0 other chains. 
      chain W     have identified ncs to     0 other chains. 
     For .Gelly cards produced see below - after pruning
 
 Setting up BUSTER_SET groups
 Have found    89 BUSTER_SET cards
 Now silently applying automatic/dictionary set definitions. For a verbose listing use '-verbose_set' flag.
 Applying user defined set definitions:
 =====NOTE BUSTER_SET ncsautoXcld = Water --> set 'ncsautoXcld' now has   966 atoms 
 =====NOTE BUSTER_SET ncsautoABset = Chain_A \ ncsautoXcld --> set 'ncsautoABset' now has  2781 atoms 
 Have defined    49 sets: 
      set:                 'All' has   6517 atoms in   1667 residues - automatically defined
      set:               'Empty' has      0 atoms in      0 residues - automatically defined
      set:            'ExoticAA' has      0 atoms in      0 residues - defined by dictionary file
      set:           'ExoticNUC' has      0 atoms in      0 residues - defined by dictionary file
      set:              'NotHET' has   5514 atoms in    698 residues - defined by dictionary file
      set:             'Chain_A' has   2781 atoms in    351 residues - automatically defined
      set:             'Chain_B' has   2770 atoms in    350 residues - automatically defined
      set:             'Chain_S' has    567 atoms in    567 residues - automatically defined
      set:             'Chain_W' has    399 atoms in    399 residues - automatically defined
      set:               'Water' has    966 atoms in    966 residues - defined by dictionary file
      set:          'StdProtein' has   5514 atoms in    698 residues - defined by dictionary file
      set:             'Protein' has   5514 atoms in    698 residues - defined by dictionary file
      set:                'Back' has   2794 atoms in    698 residues - defined by dictionary file
      set:                'Side' has   2720 atoms in    650 residues - defined by dictionary file
      set:        'RnaCanonical' has      0 atoms in      0 residues - defined by dictionary file
      set:        'DnaCanonical' has      0 atoms in      0 residues - defined by dictionary file
      set:     'RnaDnaCanonical' has      0 atoms in      0 residues - defined by dictionary file
      set:     'DnaRnaCanonical' has      0 atoms in      0 residues - defined by dictionary file
      set:         'RnaExtended' has      0 atoms in      0 residues - defined by dictionary file
      set:         'DnaExtended' has      0 atoms in      0 residues - defined by dictionary file
      set:      'RnaDnaExtended' has      0 atoms in      0 residues - defined by dictionary file
      set:      'DnaRnaExtended' has      0 atoms in      0 residues - defined by dictionary file
      set:                 'Dna' has      0 atoms in      0 residues - defined by dictionary file
      set:                 'Rna' has      0 atoms in      0 residues - defined by dictionary file
      set:              'RnaDna' has      0 atoms in      0 residues - defined by dictionary file
      set:              'DnaRna' has      0 atoms in      0 residues - defined by dictionary file
      set:             'Nucleic' has      0 atoms in      0 residues - defined by dictionary file
      set:             'Polymer' has   5514 atoms in    698 residues - defined by dictionary file
      set: 'BufferWeichenberger' has     26 atoms in      2 residues - defined by dictionary file
      set:    'BufferAdditional' has      0 atoms in      0 residues - defined by dictionary file
      set:              'Buffer' has     26 atoms in      2 residues - defined by dictionary file
      set:         'MetalAlkali' has      0 atoms in      0 residues - defined by dictionary file
      set:  'MetalAlkalineEarth' has      0 atoms in      0 residues - defined by dictionary file
      set:     'MetalTransition' has      0 atoms in      0 residues - defined by dictionary file
      set: 'MetalPostTransition' has      0 atoms in      0 residues - defined by dictionary file
      set:    'MetalLanthanides' has      0 atoms in      0 residues - defined by dictionary file
      set:               'Metal' has      0 atoms in      0 residues - defined by dictionary file
      set:         'SugarAlphaD' has      0 atoms in      0 residues - defined by dictionary file
      set:         'SugarAlphaL' has      0 atoms in      0 residues - defined by dictionary file
      set:               'Sugar' has      0 atoms in      0 residues - defined by dictionary file
      set:               'Other' has     37 atoms in      3 residues - defined by dictionary file
      set:            'Hydrogen' has      0 atoms in      0 residues - defined by dictionary file
      set:         'nonHydrogen' has   6517 atoms in   1667 residues - defined by dictionary file
      set:             'OccZero' has      0 atoms in      0 residues - defined by dictionary file
      set:          'OccNonZero' has   6517 atoms in   1667 residues - defined by dictionary file
      set:             'OccFull' has   6474 atoms in   1664 residues - defined by dictionary file
      set:          'OccNonFull' has     43 atoms in      3 residues - defined by dictionary file
      set:         'ncsautoXcld' has    966 atoms in    966 residues - user specified
      set:        'ncsautoABset' has   2781 atoms in    351 residues - user specified
 No command line arguments '-occ_allhet' or '-occ_restyp' so will not setup automated occcupancy refinement
 
 Interpreting CONSTANT and FREE cards: 
 =====CONSTANT     OCC
 Have found and interpreted      1 CONSTANT cards in the TNT input. 
 Before any COMBINE - Number of atoms with fixed XYZ =     0
 Before any COMBINE - Number of atoms with fixed B   =     0
 Before any COMBINE - Number of atoms with fixed OCC =  6517
 Before any COMBINE - Number of atoms with fixed BIJ =   966
                            cf total number of atoms =  6517
 
 There are no COMBINE cards to be found in the TNT card input 
 
 There are no NOTE BUSTER_COMBINE_RES cards to be found in the TNT card input 

 ############### Working out full connectivity list


 ############### Working out equivalent atoms


 Residue with most atoms given below:
        A|58:N(TRP)         
        A|58:CA(TRP)        
        A|58:CB(TRP)        
        A|58:CG(TRP)        
        A|58:CD1(TRP)       
        A|58:NE1(TRP)       
        A|58:CE2(TRP)       
        A|58:CD2(TRP)       
        A|58:CE3(TRP)       
        A|58:CZ3(TRP)       
        A|58:CH2(TRP)       
        A|58:CZ2(TRP)       
        A|58:C(TRP)         
        A|58:O(TRP)         


 Have no '-equiv_check_plus' argument - so will take atom type into
 account when checking for equivalence of otherwise identical atoms.
 

 ############### Looking at dihedrals


 ############### Looking at chirals

 NOTE BUSTER_CHIRAL_ANGLECUT card found - read value=    -31.00 degs from it
 NOTE BUSTER_CHIRAL_SIGMA card found - read value=      1.70 degs from it
 RANGE allowed for B variables in refinement:   from      3.00000 to    300.00000

 ############### Preparing for PDB output


 ############### Processing extra restraints explicitly defined

 No 'NOTE BUSTER_TARGET' cards or -target?? args found (used to read coordinates files for soft ncs to external)
 No 'NOTE BUSTER_OCCSUM'     cards found - so not using sum of occupancy restraints
 No 'NOTE BUSTER_DISTANCE'   cards found - so not setting up any additional atom-atom distance restraints
 No 'NOTE BUSTER_UTILANGLE'  cards found - so not setting up any additional restraints on any angle
 No 'NOTE BUSTER_UTILTOR'    cards found - so not setting up any additional sinusoidal restraints on torsions
 
 --- Have some similarity definitions 
     Similarity restraints on RMSD use superposition routines from: 
     Coutsias, E.A., Seok, C., Dill, K.A.(2004)
     "Using quaternions to calculate RMSD", J. Comput. Chem., 25:1849-1857 

 Have found    1 'NOTE BUSTER_SIM_DEFINE' cards 
     Maximum number of chains involved in any SIM group (excluding the template)=     1


 ########## Interpreting card: NOTE BUSTER_SIM_DEFINE ncsautoAB ncsautoABset B
     Number of atoms in template set=  2781
     Number of related chains (other than template) in SIM set=    1
     List of chains to be restrained (excluding template)=   B   
     Pairwise RMSD, XYZ in Angstroms, for SIM group: ncsautoAB
                 A         B   
     A         -----     ----- 
     B         0.634     ----- 
     Overall RMSD,XYZ for the SIM group is     0.634 Angstroms
     Pairwise rms deviation of [individual B's minus their set/chain average] in Angs**2
                 A         B         Bavg
     A         -----     -----    113.87
     B         55.37     -----     72.47
     Overall RMSD,B   for the SIM group is    55.374 Angs**2
     The number of common atoms for the above fits:
                 A         B   
     A         -----     ----- 
     B          2770     ----- 
     (compared to the template set that has    2781 atoms in it)
     List of atoms that are absent in one or more chains for this group: 
                                    A    B   
                    C01     501     +    -
                    C02     501     +    -
                    O03     501     +    -
                    N04     501     +    -
                    C05     501     +    -
                    C06     501     +    -
                    C07     501     +    -
                    N08     501     +    -
                    C09     501     +    -
                    C10     501     +    -
                    O11     501     +    -
     (key  +  atom present for this chain 
           -  atom absent  for this chain)
 transforms for each of the superpositions orthogonal matrix r11 r12 r13 r21 r22 r23 r31 r32 r33 tx ty tz (PDBSET syntax): 
TRANS_AB -0.872851 -0.487076 -0.029812 -0.486968  0.873347 -0.011249  0.031515  0.004699 -0.999492    33.917    33.132    14.540
 reverse transforms 
TRANS_BA -0.872851 -0.486968  0.031515 -0.487076  0.873347  0.004699 -0.029812 -0.011249 -0.999492    45.280   -12.484    15.916
 
 Setting up LSSR interatomic distance restraints
 Interpreting card: NOTE BUSTER_SIM_RESTRAIN_LSSR ncsautoAB 6.666
        setting up interatomic distance pair restraints with:
        Dmin=         0.00
        Dmax=         5.50
        sigma=        0.20
        Vmax=         3.00
        weight=       6.67
        number of distance pairs defined for this set is      35023
 Have not supplied either '-sim_swap_equiv' or '-sim_swap_equiv_plus' argument 
   so will not try swaping equivalent atoms to improve NCS and/or target agreement.
 
 NCS by LSSR outlier analysis
 -autoncs option automatic NCS prune routine -------- 
 Residues pruned out by gradient condition:
           A|27      A|30      A|34      A|54      A|87     A|135     A|148
          A|149     A|150     A|151     A|152     A|181     A|182     A|184
          A|185     A|186     A|187     A|189     A|216     A|217     A|219
          A|220     A|250     A|253     A|255     A|259     A|264     A|310
          A|319     A|328     A|344     A|345     A|401      B|27      B|30
           B|34      B|54      B|87     B|135     B|148     B|149     B|150
          B|151     B|152     B|181     B|182     B|184     B|185     B|186
          B|187     B|189     B|216     B|217     B|219     B|220     B|250
          B|253     B|255     B|259     B|264     B|310     B|319     B|328
          B|344     B|345     B|401
  
 Residues pruned out by function/max condition:
          A|149!    A|187!    A|188     A|220!    A|259!    A|264!    A|346 
         A|ENDA     A|401!    B|149!    B|187!    B|188     B|220!    B|259!
          B|264!    B|346    B|ENDA     B|401!
  
 Total number of residues for which LSSR restraints have been pruned out is       72 out of      700 participating
 Number of LSSR restraints pruned out is      5196 this is   14.8% of the total           35023
  
 -autoncs .Gelly cards suitable for adaptation for manual definition/pruning
           (note auto pruning is done in a slightly different way so exact results may differ slightly)
 -autoncs option .Gelly cards also written to file  auto_gelly_cards.txt
NOTE BUSTER_SET ncsautoXcld = Water    # card added - produced by -autoncs
NOTE BUSTER_SET ncsautoXcld = ncsautoXcld + { A|27 A|30 A|34 A|54 A|87 A|135 }    # card added - produced by -autoncs
NOTE BUSTER_SET ncsautoXcld = ncsautoXcld + { A|148 A|149 A|150 A|151 A|152 }    # card added - produced by -autoncs
NOTE BUSTER_SET ncsautoXcld = ncsautoXcld + { A|181 A|182 A|184 A|185 A|186 }    # card added - produced by -autoncs
NOTE BUSTER_SET ncsautoXcld = ncsautoXcld + { A|187 A|188 A|189 A|216 A|217 }    # card added - produced by -autoncs
NOTE BUSTER_SET ncsautoXcld = ncsautoXcld + { A|219 A|220 A|250 A|253 A|255 }    # card added - produced by -autoncs
NOTE BUSTER_SET ncsautoXcld = ncsautoXcld + { A|259 A|264 A|310 A|319 A|328 }    # card added - produced by -autoncs
NOTE BUSTER_SET ncsautoXcld = ncsautoXcld + { A|344 A|345 A|346 A|ENDA A|401 }    # card added - produced by -autoncs
NOTE BUSTER_SET ncsautoABset = Chain_A \ ncsautoXcld    # card added - produced by -autoncs
NOTE BUSTER_SIM_DEFINE ncsautoAB ncsautoABset B    # card added - produced by -autoncs
NOTE BUSTER_SIM_RESTRAIN_LSSR ncsautoAB 6.666    # card added - produced by -autoncs

 LSSR function contribution before pruning     44789.3
                             after pruning     11500.9
 Graph of which residues pruned from LSSR: ncs_pruned.mtv
 Graph of LSSR function outliers ncs_function_outliers.mtv
 Graph of LSSR gradient outliers ncs_gradient_outliers.mtv
 LSSR - analysis of distance difference distribution for NCS
         DeltaD less than 0.5 Angs:  proportion of population= 98.62% average DeltaD of subset     0.014 Angs
 Histogram of LSSR deltaD distribution ncs_histogram.mtv
 
 Interpreting card: NOTE BUSTER_TLS_SET tls1 { A|* }
 Interpreting card: NOTE BUSTER_TLS_SET tls2 { B|* }
 Interpreting card: NOTE BUSTER_TLS_SET tls1 { A|* }
 Interpreting card: NOTE BUSTER_TLS_T   tls1     -9.27022E-02  3.86691E-02  3.74474E-02 -4.12978E-02  3.98790E-02 -2.98646E-02
 Interpreting card: NOTE BUSTER_TLS_L   tls1      1.77308E+00  1.37785E+00  1.02337E+00 -5.24463E-01 -1.21175E+00  5.33191E-01
 Interpreting card: NOTE BUSTER_TLS_S   tls1      9.10334E-02 -3.37935E-01  3.79187E-01 -3.08938E-01  1.66879E-01 -1.72939E-01 -3.02906E-02 -2.18204E-01
 Interpreting card: NOTE BUSTER_TLS_O   tls1     -2.30832E+01 -8.41146E+01  1.12764E+01
 Interpreting card: NOTE BUSTER_TLS_SET tls2 { B|* }
 Interpreting card: NOTE BUSTER_TLS_T   tls2     -1.45544E-01 -8.72395E-02  1.24440E-01  1.38485E-02  3.79911E-02 -6.45887E-03
 Interpreting card: NOTE BUSTER_TLS_L   tls2      1.08099E+00  8.79887E-01  6.84078E-01  5.26234E-01 -7.68579E-01 -5.77148E-01
 Interpreting card: NOTE BUSTER_TLS_S   tls2     -5.61912E-02  1.02074E-01 -8.18820E-02 -1.38494E-02  1.32376E-01  4.36276E-02 -1.03695E-01  9.56923E-02
 Interpreting card: NOTE BUSTER_TLS_O   tls2      2.22817E+01 -7.23258E+01  3.51644E+00
 Interpreting card: NOTE BUSTER_TLS_FIX tls1 ALL
 Interpreting card: NOTE BUSTER_TLS_FIX tls2 ALL


 TLS information. Total number of groups:     2


 Group   Natoms    FixRB   FixTLS   Group Tag    Group Spec
     1     2781      Y        Y     tls1    { A|* }
     2     2770      Y        Y     tls2    { B|* }
 
NOTE BUSTER_TLS_SET tls1 { A|* }
NOTE BUSTER_TLS_T   tls1     -9.27022E-02  3.86691E-02  3.74474E-02 -4.12978E-02  3.98790E-02 -2.98646E-02
NOTE BUSTER_TLS_L   tls1      1.77308E+00  1.37785E+00  1.02337E+00 -5.24463E-01 -1.21175E+00  5.33191E-01
NOTE BUSTER_TLS_S   tls1      9.10334E-02 -3.37935E-01  3.79187E-01 -3.08938E-01  1.66879E-01 -1.72939E-01 -3.02906E-02 -2.18204E-01
NOTE BUSTER_TLS_O   tls1     -2.30832E+01 -8.41146E+01  1.12764E+01
NOTE BUSTER_TLS_FIX tls1 ALL
 
NOTE BUSTER_TLS_SET tls2 { B|* }
NOTE BUSTER_TLS_T   tls2     -1.45544E-01 -8.72395E-02  1.24440E-01  1.38485E-02  3.79911E-02 -6.45887E-03
NOTE BUSTER_TLS_L   tls2      1.08099E+00  8.79887E-01  6.84078E-01  5.26234E-01 -7.68579E-01 -5.77148E-01
NOTE BUSTER_TLS_S   tls2     -5.61912E-02  1.02074E-01 -8.18820E-02 -1.38494E-02  1.32376E-01  4.36276E-02 -1.03695E-01  9.56923E-02
NOTE BUSTER_TLS_O   tls2      2.22817E+01 -7.23258E+01  3.51644E+00
NOTE BUSTER_TLS_FIX tls2 ALL


 TLS: mmCIF group specification begin


#
loop_
_pdbx_refine_tls_group.id
_pdbx_refine_tls_group.refine_tls_id
_pdbx_refine_tls_group.beg_PDB_ins_code
_pdbx_refine_tls_group.beg_auth_asym_id
_pdbx_refine_tls_group.beg_auth_seq_id
_pdbx_refine_tls_group.end_PDB_ins_code
_pdbx_refine_tls_group.end_auth_asym_id
_pdbx_refine_tls_group.end_auth_seq_id
_pdbx_refine_tls_group.selection
_pdbx_refine_tls_group.selection_details
   1   1 . A   -1 . A  346 . '{ A|* }'
   2   1 . A  401 . A  501 . '{ A|* }'
   3   2 . B   -1 . B  346 . '{ B|* }'
   4   2 . B  401 . B  401 . '{ B|* }'
#


 TLS: mmCIF group specification end


 Interpreting card: NOTE BUSTER_ANISO_SET Empty
 Interpreting card: NOTE BUSTER_ANISO_WEIGHT SCA  16.0
 Interpreting card: NOTE BUSTER_ANISO_WEIGHT SPH   0.6    0.6
 Interpreting card: NOTE BUSTER_ANISO_WEIGHT SIM   7.8    4.8    2.6    0
 Interpreting card: NOTE BUSTER_ANISO_WEIGHT RIG  21.5   21.5    3.7    0


 After TLS and ANISO: Number of atoms with fixed/combined xyz =     0
 After TLS and ANISO: Number of atoms with fixed/combined B   =     0
 After TLS and ANISO: Number of atoms with fixed/combined OCC =  6517
 After TLS and ANISO: Number of atoms with fixed/combined BIJ =  6517
 After TLS and ANISO: Number of atoms in a TLS group          =  5551
                                     cf total number of atoms =  6517
 

 ############### Setup state vector

 No free_vect_ulim_xyz argument found: using default of  100.000
 No free_vect_llim_xyz argument found: using default of -100.000
 Number of parameters added for TLS description                 =       0
 Number of parameters to describe molecular conformation        =   26068

 ############### Setup free vector

 Number of free parameters to be adjusted in refinement   =   26068

 ############### Setting up own geometry


 No command line argument '-tnt_fn' so will use gelly to do geometry function calculations

 ============= Call TNT functions


 ============= Get TNT symmetry information

 
      Getting symmetry operators from TNT. 
          gelly will classify symmetry using pdb-like convention: 
               SYMOP   SYMMETRY
              NNNMMM   OPERATOR
                1555   X,Y,Z
                2555   -Y,X-Y,Z
                3555   -X+Y,-X,Z
                4555   -X,-Y,1/2+Z
                5555   Y,-X+Y,1/2+Z
                6555   X-Y,X,1/2+Z
          where NNN -> operator number and MMM -> translation vector
 

 ============= Own Gelly geometry/restraints setup


 ----------- Identify bond-types


    Setting up GEOMBONDREC_TYPE_HASH* to be able to map a given bond type (e.g.
    "single") to an internal integer (GBOND_TYPE=1) etc.

    Using default  GEOMBONDREC_TYPE_HASH_STR='SING* 1 DOUB* 2 TRIP* 3 AROM* 4 DELO* 4 METAL* 5':
    to override default use a NOTE BUSTER_GEOMBONDREC_TYPE_HASH_STR gelly card.

 ----------- Classify torsion into peptide omega and other


 ----------- Setup of non-bonded terms

 Ideal-distance contact term setup:
    Restraint function constants (normally these values are from NOTE BUSTER_IDEAL_CONTACT_* cards)
    Sigma for general contacts      0.200 Angs
    Sigma for 1-4 contacts          0.100 Angs (contacts across a torsion)
    Sigma for 1-5 contacts          0.150 Angs
    Sigma for 1-N contacts          0.200 Angs
    Sigma for HBOND pairs           0.100 Angs (contacts between HB_types A and D or B's)
    Contact distance increment for each atom in HBOND          -0.150 Angs 
    Contact distance increment for H-bond acceptor              0.100 Angs 
    Contact distance increment for 1-4 with an O atom          -0.050 Angs 
    Contact distance increment for 1-4 with an N atom          -0.180 Angs 
    Contact distance increment for 1-4 with an C atom          -0.370 Angs 
    Contact distance increment for 1-4 with an S atom          -0.150 Angs 
    Contact distance increment for 1-4 with an P atom          -0.150 Angs 
    Contact distance increment for 1-4 with any other atom     -0.150 Angs 
    All atoms matched for CCP4 atom type.
    Number of CCP4 atom types used is         24 (H-bound and not H-bound counted separately)

 Setting up short/ideal-distance contacts
   Using gelly special position handling. (For TNT instead rerun with argument '-special_tnt')

   No command line argument '-special_all' specified. We will auto-generate
   EXCLUDE cards only for atom-atom contacts across symmetry (i.e. on special
   position) when this involves a contact within a residue to itself.

   Have set up non-bonded exclusions - 1-3 contacts
   Atom A|27:CA         makes the most 1-3 contacts:  6

   Have set up non-bonded exclusions - 1-4 contacts
   Atom A|27:N          makes the most 1-4 contacts:  7

   Have set up non-bonded exclusions - 1-5 contacts
   Atom A|28:C.A        makes the most 1-5 contacts: 10

 ############################################################################################################
 ###################################################### setting up contact terms
 ############################################################################################################


 ============= Call Gelly versions of TNT functions

 WARNING: 1-4 contact without 1-(2,3)-4 data for A|401:C2(CIT)        and A|401:O6(CIT)        X-X
 WARNING: 1-4 contact without 1-(2,3)-4 data for A|401:O7(CIT)        and A|401:C5(CIT)        X-X
 WARNING: 1-4 contact without 1-(2,3)-4 data for A|401:O7(CIT)        and A|401:O5(CIT)        X-X
 WARNING: 1-4 contact without 1-(2,3)-4 data for A|401:C4(CIT)        and A|401:O6(CIT)        X-X
 WARNING: 1-4 contact without 1-(2,3)-4 data for A|501:C01(LIG)       and A|501:C05(LIG)       X-X
 WARNING: 1-4 contact without 1-(2,3)-4 data for A|501:C06(LIG)       and A|501:C09(LIG)       X-X
 WARNING: 1-4 contact without 1-(2,3)-4 data for A|501:C07(LIG)       and A|501:C10(LIG)       X-X
 WARNING: 1-4 contact without 1-(2,3)-4 data for B|401:C1(CIT)        and B|401:C4(CIT)        X-X
 WARNING: 1-4 contact without 1-(2,3)-4 data for B|401:C2(CIT)        and B|401:O6(CIT)        X-X
 WARNING: 1-4 contact without 1-(2,3)-4 data for B|401:O7(CIT)        and B|401:O5(CIT)        X-X
 WARNING: 1-4 contact without 1-(2,3)-4 data for B|401:C4(CIT)        and B|401:O6(CIT)        X-X

       TERM      weight      number      TNT_fn    Gelly_fn        diff
       BOND       2.000        5665    1713.324    1713.324      -0.000
       ANGL       2.000        7687    5283.260    5283.260       0.000
       IMPR       0.000           0       0.000       0.000       0.000
       TORS       2.000        1973    3251.478    3251.478      -0.000
       TRIG       2.000           0       0.000       0.000       0.000
       PLAN       5.000         967    2449.119    2449.119       0.000
       CONT       5.000           0     150.711       0.000     150.711
       BCOR      10.000        5630    8759.750   17019.895   -8260.146


 ============= Call Gelly geometry functions

   Switching torsion functional form to sinusoidal rather than split harmonic functional.
      if you want this not to happen then rerun with '-torsharm'
      Weight for sinusoidal torsion=     2.000
   BCOR function/gradient will be split between H and non-H atoms (as for any atom pair). Use '-bcorrel_all_in_hydrogens' to change this.

 ----------- Fix B-correlation restraints

 
 Checking for bonds that do not have a bcorrel restraint defined
 We will wipe all existing BCORrels if OCC=0.0 atom(s) is/are involved. This can be' switched off via the '-bcorrel_nowipe_zeroocc' flag.
 We will wipe all existing BCORrels if only non-hydrogen atom(s) with fixed B(iso) are involved. This can be switched off via the '-bcorrel_nowipe_fixb_nonhydrogens' flag.
 We will keep all existing BCORrels if hydrogen atom(s) with fixed B(iso) is/are involved. This can be switched off via the '-bcorrel_wipe_fixb_hydrogens' flag.
 No -bcorrel_missing_sigma found. So will set sigma to     2.5 Ang**2 for any bond with no existing BCORrel
 No -bcorrel_missing_sigma_hydrogens found. So will set sigma to     1.0 Ang**2 for any bond with no existing BCORrel involving hydrogens

 No active BCORels found involving OCC=0.0 atom(s)


 No active BCORels found involving fixed-B (only) non-hydrogen atom(s)

    add a default BCOR for bond A|401:C1(CIT) A|401:C2(CIT)
    add a default BCOR for bond A|401:C1(CIT) A|401:O1(CIT)
    add a default BCOR for bond A|401:C1(CIT) A|401:O2(CIT)
    add a default BCOR for bond A|401:C2(CIT) A|401:C3(CIT)
    add a default BCOR for bond A|401:C3(CIT) A|401:C4(CIT)
    add a default BCOR for bond A|401:C3(CIT) A|401:C6(CIT)
    add a default BCOR for bond A|401:C3(CIT) A|401:O7(CIT)
    add a default BCOR for bond A|401:C4(CIT) A|401:C5(CIT)
    add a default BCOR for bond A|401:C5(CIT) A|401:O3(CIT)
    add a default BCOR for bond A|401:C5(CIT) A|401:O4(CIT)
    add a default BCOR for bond A|401:C6(CIT) A|401:O5(CIT)
    add a default BCOR for bond A|401:C6(CIT) A|401:O6(CIT)
    add a default BCOR for bond A|501:C01(LIG) A|501:C02(LIG)
    add a default BCOR for bond A|501:C02(LIG) A|501:O03(LIG)
    add a default BCOR for bond A|501:C02(LIG) A|501:N04(LIG)
    add a default BCOR for bond A|501:N04(LIG) A|501:C05(LIG)
    add a default BCOR for bond A|501:C05(LIG) A|501:C06(LIG)
    add a default BCOR for bond A|501:C06(LIG) A|501:C07(LIG)
    add a default BCOR for bond A|501:C06(LIG) A|501:O11(LIG)
    add a default BCOR for bond A|501:C07(LIG) A|501:N08(LIG)
    add a default BCOR for bond A|501:N08(LIG) A|501:C09(LIG)
    add a default BCOR for bond A|501:C09(LIG) A|501:C10(LIG)
    add a default BCOR for bond A|501:C10(LIG) A|501:O11(LIG)
    add a default BCOR for bond B|401:C1(CIT) B|401:C2(CIT)
    add a default BCOR for bond B|401:C1(CIT) B|401:O1(CIT)
    add a default BCOR for bond B|401:C1(CIT) B|401:O2(CIT)
    add a default BCOR for bond B|401:C2(CIT) B|401:C3(CIT)
    add a default BCOR for bond B|401:C3(CIT) B|401:C4(CIT)
    add a default BCOR for bond B|401:C3(CIT) B|401:C6(CIT)
    add a default BCOR for bond B|401:C3(CIT) B|401:O7(CIT)
    add a default BCOR for bond B|401:C4(CIT) B|401:C5(CIT)
    add a default BCOR for bond B|401:C5(CIT) B|401:O3(CIT)
    add a default BCOR for bond B|401:C5(CIT) B|401:O4(CIT)
    add a default BCOR for bond B|401:C6(CIT) B|401:O5(CIT)
    add a default BCOR for bond B|401:C6(CIT) B|401:O6(CIT)

 Have added a total of        35 BCORrels for bonds which do not already have a BCORrel


 No bonds (for potential BCORrel addition) were skipped due to both atoms having constant/fixed B(iso)


 ----------- Deactivate hydrogen-free planes if hydrogen atoms present

 A total of       0 planes have been deactivated because they are duplicated hydrogen-free and with-hydrogen forms.

 ----------- Handle peptide omega angle(s)

      Torsion angles split into peptide omega and other
         Number of peptide omega angles is       698
         Number of other torsion is             1275
      Peptide omega angles are monitored rather than directly restrained (PLANE used instead).

 ----------- Fix cis-PRO

 Now apply the fix to get correct restraints for cis PRO residues
   Have got      11 bonds records and      26 angles
   from GEOMETRY cards for the special PROC residue
   Weight for ideal-distance contact term=     4.000

 ----------- Prepare SSBOND/LINK for output PDB file


 ----------- Prepare for QM


 ----------- Prepare symmetry in utility distance restraints


 ############### Setting up Rhofit contacts (if needed)

 No ligand-(fixed protein) contact term will be used

 ############### Working out initial function and gradient


   MaxLik Scaling Cycle 0002
 
 Initial functional value = 4.790609E+05 rms gradient=      98.8473
 X-ray contribution to function value = 4.263307E+05
 Geometry contribution (inc ncs)      =      52730.1
 Geometry function summary
     term                           number     weight       rms        GooF      function     fn/numb
     ================================================================================================
     BOND bond lengths (angs)       5665       2.000      0.0078       0.389      1713.32       0.302
     ANGL bond angles (degs)        7687       2.000        0.98       0.586      5283.26       0.687
     TORS split-harmonic tors       1275       0.000       17.96       1.129         0.00       0.000
     SINTOR sinusoidal tors         1275       2.000       17.96                  2436.00       1.911
     PLAN general plane (angs)       967       5.000      0.0142       0.712      2449.12       2.533
     BCORrelations (angs^2)         5665      10.000       1.537       0.549     17047.06       3.009
     CONT (bad contacts)               0       5.000                                 0.00
     IDEAL(ideal-dist contact)      5673       4.000                             12254.62
     CHIRAL (gelly semiharmon)       740       5.000                                45.87
     SIM similarity restraint for NCS and/or TARGET                              11500.89
     OCCSUM restraint on sum of occupanies                                           0.00       as none defined
     DISTAN utility restraints interatomic distance                                  0.00       as none defined
     UTILANGLE utility angle restraints                                              0.00       as none defined
     UTILTOR utility restraints on torsion angles                                    0.00       as none defined
     Peptide omega torsion angles                           2.88 degs - monitor only

 Weighted rms      0.0078         0.984  (temporary rms bond and angle for BUSTER to pick up!)
 Weighted intra-residual rms(bond) for StdProtein (excluding H)      0.0076
 Weighted intra-residual rms(bond) for Buffer     (excluding H)      0.0032

 ############### Running initial check


 Initial Geometry Sanity Check

 Check for duplicate bonds: no duplicates found
 
 Check for duplicate or missing bond angles: all are OK
 Check for bond between an atom and itself not involving symmetry:  all are OK
   There are no bad contacts where Delta/sigma is greater than     6.5
   Worst contact dist=   2.990 std=   3.802 sigma=   0.206 abs(Delta/sigma)=    3.941 ats A|104:CE (MET) B|23:CB (PRO)
   It is likely that the geometry libraries, sequence info and links match the input molecule
 GEOMETRY CHECK OVERALL:  PASS


 ############### Creating geometry/screen report


 ############### Option "tao" - run Tao optimizer

 TAO: Toolkit for Advanced Optimization. Reference: 
     Munson, T., Sarich, J., Wild, S., Benson, S., McInnes, L.C. (2018)
     "TAO User Manual (Revision 3.9)", Mathematics and Computer Science
     Division, Argonne National Laboratory
     ANL/MCS-TM-322, http://www.mcs.anl.gov/petsc/
 
 Have found -keeppdb flag so will write out a .pdb file every 1000000 its during opt
 Will apply TAO optimization 
 Maximum number of iterations      100
 Convergence test on Grms       4.0000 (-glim)
 Limit on rms displacement to original position (rmsD2init): NONE test inactive (-dlim)

    analysing argument no 0 : -autoncs with j=0
    analysing argument no 1 : -hydrogens_input_occ2zero with j=0
    analysing argument no 2 : -hydrogens_output_occ2heavy with j=0
    analysing argument no 3 : -screen with j=0
    analysing argument no 4 : 100 with j=0
    analysing argument no 5 : -screen_sigma with j=0
    analysing argument no 6 : 3.0 with j=0
    analysing argument no 7 : -keeppdb with j=0
    analysing argument no 8 : 1000000 with j=0
    analysing argument no 9 : -taomonitor_verbose with j=0
    found argument starting with "-tao" : -taomonitor_verbose
    analysing argument no 10 : 2 with j=1
    found value after -tao/-options_/-viewer_ argument : "2"
 PETSC/TAO: setting option "-taomonitor_verbose" = "2"


   MaxLik Scaling Cycle 0003
   Iters     Tot_Funct    Xray_Funct    Geom_Funct          Grms     rmsD2init     maxD2init             R         Rfree           LLG       LLGfree     rms(bond)    rms(angle)       Opttag_H
   Iters             K   B_IMPF_FRAG        K_MISS   B_TEMP_MISS   K_IMPF_MISS   B_IMPF_MISS        K_SOLV   B_TEMP_SOLV   K_IMPF_SOLV   B_IMPF_SOLV        B_TEMP     B_TEMP_11     B_TEMP_12     B_TEMP_22     B_TEMP_13     B_TEMP_23     B_TEMP_33      K_WILSON      B_WILSON      FGCYCLES       Scatag_H
   Iters          BOND         ANGLE      IMPROPER       TORSION    SINTORSION      TRIGONAL        PLANAR  BCORRELATION       CONTACT     IDEALDIST        CHIRAL    SIMILARITY       OCCSSUM  UTILDISTANCE     UTILANGLE   UTILTORSION        RHOFIT        ADP-01        ADP-02        ADP-03        ADP-04        ADP-05        ADP-06        ADP-07        ADP-08        ADP-09        ADP-10      RHOFITPC            QM       Funtag_H
       0  4.789799E+05  4.263282E+05       52651.7       96.2444  0.000000E+00  0.000000E+00      0.189177      0.240092  0.000000E+00  0.000000E+00    0.00779694      0.983684       Opttag_D
       0       82.0777       1.45246       1.00000  0.000000E+00       1.00000  0.000000E+00      0.941742       96.7280       1.00000       39.6585  0.000000E+00       9.27427  0.000000E+00       9.27427  0.000000E+00  0.000000E+00      -18.5485       83.8070       4.12606        2       Scatag_D
       0       1713.32       5283.26  0.000000E+00  0.000000E+00       2436.00  0.000000E+00       2449.12       16968.6  0.000000E+00       12254.6       45.8697       11500.9 -1.000000E+10 -1.000000E+10 -1.000000E+10 -1.000000E+10 -1.000000E+10  0.000000E+00  0.000000E+00  0.000000E+00  0.000000E+00  0.000000E+00  0.000000E+00  0.000000E+00  0.000000E+00  0.000000E+00  0.000000E+00  0.000000E+00  0.000000E+00       Funtag_D
   Geometry Restraint Deviations SCREEN Information

 

   MaxLik Scaling Cycle 0004

   MaxLik Scaling Cycle 0005

   MaxLik Scaling Cycle 0006

   Wilson plot

   Statistics in resolution bins after refinement cycle 0001
```


|  |  |
| --- | --- |
|  | - Rxpct factors plot- Rxpct factors table- <Log-Lik Gain>- Ln Sigma A- Correlation Coefficients |


```
   Iters     Tot_Funct    Xray_Funct    Geom_Funct          Grms     rmsD2init     maxD2init             R         Rfree           LLG       LLGfree     rms(bond)    rms(angle)
   Iters             K   B_IMPF_FRAG        K_MISS   B_TEMP_MISS   K_IMPF_MISS   B_IMPF_MISS        K_SOLV   B_TEMP_SOLV   K_IMPF_SOLV   B_IMPF_SOLV        B_TEMP     B_TEMP_11     B_TEMP_12     B_TEMP_22     B_TEMP_13     B_TEMP_23     B_TEMP_33      K_WILSON      B_WILSON      FGCYCLES
   Iters          BOND         ANGLE      IMPROPER       TORSION    SINTORSION      TRIGONAL        PLANAR  BCORRELATION       CONTACT     IDEALDIST        CHIRAL    SIMILARITY       OCCSSUM  UTILDISTANCE     UTILANGLE   UTILTORSION        RHOFIT        ADP-01        ADP-02        ADP-03        ADP-04        ADP-05        ADP-06        ADP-07        ADP-08        ADP-09        ADP-10      RHOFITPC            QM
       1  4.770390E+05  4.261774E+05       50861.7       71.9315  4.796924E-04    0.00388047      0.189155      0.240287  3.949094E-04 -7.731307E-04    0.00776940      0.983751       Opttag_D
       1       82.0434       1.44404       1.00000  0.000000E+00       1.00000  0.000000E+00      0.941823       96.5347       1.00000       39.6265  0.000000E+00       9.28768  0.000000E+00       9.28768  0.000000E+00  0.000000E+00      -18.5754       83.8070       4.12606        3       Scatag_D
       1       1701.24       5283.99  0.000000E+00  0.000000E+00       2436.08  0.000000E+00       2448.08       15201.9  0.000000E+00       12256.4       46.1798       11487.8 -1.000000E+10 -1.000000E+10 -1.000000E+10 -1.000000E+10 -1.000000E+10  0.000000E+00  0.000000E+00  0.000000E+00  0.000000E+00  0.000000E+00  0.000000E+00  0.000000E+00  0.000000E+00  0.000000E+00  0.000000E+00  0.000000E+00  0.000000E+00       Funtag_D
   Geometry Restraint Deviations SCREEN Information

 

   MaxLik Scaling Cycle 0007

   Wilson plot

   Statistics in resolution bins after refinement cycle 0002
```


|  |  |
| --- | --- |
|  | - Rxpct factors plot- Rxpct factors table- <Log-Lik Gain>- Ln Sigma A- Correlation Coefficients |


```
   Iters     Tot_Funct    Xray_Funct    Geom_Funct          Grms     rmsD2init     maxD2init             R         Rfree           LLG       LLGfree     rms(bond)    rms(angle)
   Iters             K   B_IMPF_FRAG        K_MISS   B_TEMP_MISS   K_IMPF_MISS   B_IMPF_MISS        K_SOLV   B_TEMP_SOLV   K_IMPF_SOLV   B_IMPF_SOLV        B_TEMP     B_TEMP_11     B_TEMP_12     B_TEMP_22     B_TEMP_13     B_TEMP_23     B_TEMP_33      K_WILSON      B_WILSON      FGCYCLES
   Iters          BOND         ANGLE      IMPROPER       TORSION    SINTORSION      TRIGONAL        PLANAR  BCORRELATION       CONTACT     IDEALDIST        CHIRAL    SIMILARITY       OCCSSUM  UTILDISTANCE     UTILANGLE   UTILTORSION        RHOFIT        ADP-01        ADP-02        ADP-03        ADP-04        ADP-05        ADP-06        ADP-07        ADP-08        ADP-09        ADP-10      RHOFITPC            QM
       2  4.760286E+05  4.260434E+05       49985.2       58.6254    0.00102258    0.00874997      0.189111      0.240343  7.457264E-04   -0.00116448    0.00776432      0.985011       Opttag_D
       2       82.0019       1.43560       1.00000  0.000000E+00       1.00000  0.000000E+00      0.941845       96.3605       1.00000       39.5780  0.000000E+00       9.30185  0.000000E+00       9.30185  0.000000E+00  0.000000E+00      -18.6037       83.8070       4.12606        1       Scatag_D
       2       1699.02       5297.53  0.000000E+00  0.000000E+00       2436.18  0.000000E+00       2447.27       14322.9  0.000000E+00       12259.4       46.4864       11476.5 -1.000000E+10 -1.000000E+10 -1.000000E+10 -1.000000E+10 -1.000000E+10  0.000000E+00  0.000000E+00  0.000000E+00  0.000000E+00  0.000000E+00  0.000000E+00  0.000000E+00  0.000000E+00  0.000000E+00  0.000000E+00  0.000000E+00  0.000000E+00       Funtag_D
   Geometry Restraint Deviations SCREEN Information

 
       3  4.753994E+05  4.259059E+05       49493.5       54.5908    0.00160172     0.0138078      0.189068      0.240395    0.00110586   -0.00152939    0.00777128      0.987860       Opttag_D
       3       81.9561       1.42668       1.00000  0.000000E+00       1.00000  0.000000E+00      0.941842       96.1826       1.00000       39.5272  0.000000E+00       9.31736  0.000000E+00       9.31736  0.000000E+00  0.000000E+00      -18.6347       83.8070       4.12606        1       Scatag_D
       3       1702.07       5328.21  0.000000E+00  0.000000E+00       2436.47  0.000000E+00       2446.98       13801.0  0.000000E+00       12263.1       46.7233       11469.0 -1.000000E+10 -1.000000E+10 -1.000000E+10 -1.000000E+10 -1.000000E+10  0.000000E+00  0.000000E+00  0.000000E+00  0.000000E+00  0.000000E+00  0.000000E+00  0.000000E+00  0.000000E+00  0.000000E+00  0.000000E+00  0.000000E+00  0.000000E+00       Funtag_D
       4  4.749168E+05  4.257279E+05       49188.8       54.7057    0.00237912     0.0208241      0.189020      0.240468    0.00157186   -0.00200429    0.00779232      0.988488       Opttag_D
       4       81.8920       1.41471       1.00000  0.000000E+00       1.00000  0.000000E+00      0.941823       95.9403       1.00000       39.4599  0.000000E+00       9.33997  0.000000E+00       9.33997  0.000000E+00  0.000000E+00      -18.6799       83.8070       4.12606        1       Scatag_D
       4       1711.30       5334.99  0.000000E+00  0.000000E+00       2436.94  0.000000E+00       2447.51       13478.5  0.000000E+00       12269.1       46.9316       11463.5 -1.000000E+10 -1.000000E+10 -1.000000E+10 -1.000000E+10 -1.000000E+10  0.000000E+00  0.000000E+00  0.000000E+00  0.000000E+00  0.000000E+00  0.000000E+00  0.000000E+00  0.000000E+00  0.000000E+00  0.000000E+00  0.000000E+00  0.000000E+00       Funtag_D
       5  4.745256E+05  4.256265E+05       48899.1       40.5521    0.00279135     0.0248471      0.188993      0.240506    0.00183756   -0.00230837    0.00778659      0.985968       Opttag_D
       5       81.8540       1.40783       1.00000  0.000000E+00       1.00000  0.000000E+00      0.941793       95.8070       1.00000       39.4226  0.000000E+00       9.35360  0.000000E+00       9.35360  0.000000E+00  0.000000E+00      -18.7072       83.8070       4.12606        1       Scatag_D
       5       1708.78       5307.82  0.000000E+00  0.000000E+00       2437.11  0.000000E+00       2448.32       13217.7  0.000000E+00       12271.6       46.8964       11460.7 -1.000000E+10 -1.000000E+10 -1.000000E+10 -1.000000E+10 -1.000000E+10  0.000000E+00  0.000000E+00  0.000000E+00  0.000000E+00  0.000000E+00  0.000000E+00  0.000000E+00  0.000000E+00  0.000000E+00  0.000000E+00  0.000000E+00  0.000000E+00       Funtag_D
       6  4.740373E+05  4.254575E+05       48579.8       41.7225    0.00354630     0.0320396      0.188955      0.240572    0.00228012   -0.00289426    0.00779914      0.988027       Opttag_D
       6       81.7850       1.39583       1.00000  0.000000E+00       1.00000  0.000000E+00      0.941725       95.5723       1.00000       39.3585  0.000000E+00       9.37799  0.000000E+00       9.37799  0.000000E+00  0.000000E+00      -18.7560       83.8070       4.12606        1       Scatag_D
       6       1714.29       5330.02  0.000000E+00  0.000000E+00       2437.77  0.000000E+00       2450.55       12866.4  0.000000E+00       12276.1       46.7794       11457.9 -1.000000E+10 -1.000000E+10 -1.000000E+10 -1.000000E+10 -1.000000E+10  0.000000E+00  0.000000E+00  0.000000E+00  0.000000E+00  0.000000E+00  0.000000E+00  0.000000E+00  0.000000E+00  0.000000E+00  0.000000E+00  0.000000E+00  0.000000E+00       Funtag_D
       7  4.736927E+05  4.252543E+05       48438.4       70.8271    0.00468683     0.0435241      0.188918      0.240679    0.00281230   -0.00383057    0.00785866       1.00171       Opttag_D
       7       81.6842       1.37957       1.00000  0.000000E+00       1.00000  0.000000E+00      0.941611       95.2332       1.00000       39.2734  0.000000E+00       9.41178  0.000000E+00       9.41178  0.000000E+00  0.000000E+00      -18.8236       83.8070       4.12606        1       Scatag_D
       7       1740.56       5478.63  0.000000E+00  0.000000E+00       2438.66  0.000000E+00       2455.36       12535.9  0.000000E+00       12283.4       46.7410       11459.1 -1.000000E+10 -1.000000E+10 -1.000000E+10 -1.000000E+10 -1.000000E+10  0.000000E+00  0.000000E+00  0.000000E+00  0.000000E+00  0.000000E+00  0.000000E+00  0.000000E+00  0.000000E+00  0.000000E+00  0.000000E+00  0.000000E+00  0.000000E+00       Funtag_D
       8  4.732995E+05  4.251655E+05       48133.9       41.3367    0.00528359     0.0506283      0.188912      0.240743    0.00304487   -0.00434134    0.00782706      0.989146       Opttag_D
       8       81.6292       1.37139       1.00000  0.000000E+00       1.00000  0.000000E+00      0.941549       95.0473       1.00000       39.2304  0.000000E+00       9.42895  0.000000E+00       9.42895  0.000000E+00  0.000000E+00      -18.8579       83.8070       4.12606        1       Scatag_D
       8       1726.59       5342.10  0.000000E+00  0.000000E+00       2439.27  0.000000E+00       2458.57       12378.7  0.000000E+00       12286.7       46.7864       11455.2 -1.000000E+10 -1.000000E+10 -1.000000E+10 -1.000000E+10 -1.000000E+10  0.000000E+00  0.000000E+00  0.000000E+00  0.000000E+00  0.000000E+00  0.000000E+00  0.000000E+00  0.000000E+00  0.000000E+00  0.000000E+00  0.000000E+00  0.000000E+00       Funtag_D
       9  4.730319E+05  4.251052E+05       47926.6       37.0316    0.00573602     0.0565057      0.188913      0.240799    0.00320279   -0.00476485    0.00782829      0.988992       Opttag_D
       9       81.5866       1.36527       1.00000  0.000000E+00       1.00000  0.000000E+00      0.941500       94.9027       1.00000       39.2011  0.000000E+00       9.44114  0.000000E+00       9.44114  0.000000E+00  0.000000E+00      -18.8823       83.8070       4.12606        1       Scatag_D
       9       1727.13       5340.43  0.000000E+00  0.000000E+00       2439.87  0.000000E+00       2461.14       12171.3  0.000000E+00       12288.3       46.8900       11451.6 -1.000000E+10 -1.000000E+10 -1.000000E+10 -1.000000E+10 -1.000000E+10  0.000000E+00  0.000000E+00  0.000000E+00  0.000000E+00  0.000000E+00  0.000000E+00  0.000000E+00  0.000000E+00  0.000000E+00  0.000000E+00  0.000000E+00  0.000000E+00       Funtag_D
      10  4.727293E+05  4.250243E+05       47705.1       41.2499    0.00648405     0.0665104      0.188924      0.240897    0.00341480   -0.00543058    0.00783679      0.990760       Opttag_D
      10       81.5194       1.35600       1.00000  0.000000E+00       1.00000  0.000000E+00      0.941424       94.6698       1.00000       39.1579  0.000000E+00       9.45997  0.000000E+00       9.45997  0.000000E+00  0.000000E+00      -18.9199       83.8070       4.12606        1       Scatag_D
      10       1730.88       5359.55  0.000000E+00  0.000000E+00       2440.63  0.000000E+00       2465.52       11925.0  0.000000E+00       12290.6       47.1180       11445.7 -1.000000E+10 -1.000000E+10 -1.000000E+10 -1.000000E+10 -1.000000E+10  0.000000E+00  0.000000E+00  0.000000E+00  0.000000E+00  0.000000E+00  0.000000E+00  0.000000E+00  0.000000E+00  0.000000E+00  0.000000E+00  0.000000E+00  0.000000E+00       Funtag_D
      11  4.724829E+05  4.249255E+05       47557.4       70.5049    0.00802808     0.0872765      0.188976      0.241101    0.00367349   -0.00658325    0.00789854       1.00493       Opttag_D
      11       81.3958       1.34016       1.00000  0.000000E+00       1.00000  0.000000E+00      0.941294       94.2209       1.00000       39.0826  0.000000E+00       9.49515  0.000000E+00       9.49515  0.000000E+00  0.000000E+00      -18.9903       83.8070       4.12606        1       Scatag_D
      11       1758.27       5513.98  0.000000E+00  0.000000E+00       2442.01  0.000000E+00       2474.90       11585.9  0.000000E+00       12296.3       47.5445       11438.5 -1.000000E+10 -1.000000E+10 -1.000000E+10 -1.000000E+10 -1.000000E+10  0.000000E+00  0.000000E+00  0.000000E+00  0.000000E+00  0.000000E+00  0.000000E+00  0.000000E+00  0.000000E+00  0.000000E+00  0.000000E+00  0.000000E+00  0.000000E+00       Funtag_D
      12  4.721497E+05  4.249069E+05       47242.8       31.6663    0.00844833     0.0936320      0.188996      0.241148    0.00372220   -0.00688069    0.00785406      0.990296       Opttag_D
      12       81.3630       1.33611       1.00000  0.000000E+00       1.00000  0.000000E+00      0.941258       94.0973       1.00000       39.0629  0.000000E+00       9.50412  0.000000E+00       9.50412  0.000000E+00  0.000000E+00      -19.0082       83.8070       4.12606        1       Scatag_D
      12       1738.52       5354.53  0.000000E+00  0.000000E+00       2442.37  0.000000E+00       2477.37       11453.6  0.000000E+00       12296.8       47.6286       11431.9 -1.000000E+10 -1.000000E+10 -1.000000E+10 -1.000000E+10 -1.000000E+10  0.000000E+00  0.000000E+00  0.000000E+00  0.000000E+00  0.000000E+00  0.000000E+00  0.000000E+00  0.000000E+00  0.000000E+00  0.000000E+00  0.000000E+00  0.000000E+00       Funtag_D
      13  4.719598E+05  4.248861E+05       47073.7       25.5386    0.00883217     0.0997693      0.189009      0.241164    0.00377676   -0.00707670    0.00785130      0.990256       Opttag_D
      13       81.3352       1.33243       1.00000  0.000000E+00       1.00000  0.000000E+00      0.941230       93.9855       1.00000       39.0443  0.000000E+00       9.51204  0.000000E+00       9.51204  0.000000E+00  0.000000E+00      -19.0241       83.8070       4.12606        1       Scatag_D
      13       1737.30       5354.09  0.000000E+00  0.000000E+00       2442.53  0.000000E+00       2479.22       11290.0  0.000000E+00       12297.0       47.6321       11426.0 -1.000000E+10 -1.000000E+10 -1.000000E+10 -1.000000E+10 -1.000000E+10  0.000000E+00  0.000000E+00  0.000000E+00  0.000000E+00  0.000000E+00  0.000000E+00  0.000000E+00  0.000000E+00  0.000000E+00  0.000000E+00  0.000000E+00  0.000000E+00       Funtag_D
      14  4.717732E+05  4.248584E+05       46914.8       28.6822    0.00951693      0.110434      0.189036      0.241184    0.00384909   -0.00739180    0.00785975      0.991526       Opttag_D
      14       81.2887       1.32643       1.00000  0.000000E+00       1.00000  0.000000E+00      0.941183       93.7960       1.00000       39.0130  0.000000E+00       9.52493  0.000000E+00       9.52493  0.000000E+00  0.000000E+00      -19.0499       83.8070       4.12606        1       Scatag_D
      14       1741.04       5367.83  0.000000E+00  0.000000E+00       2442.90  0.000000E+00       2482.54       11117.6  0.000000E+00       12297.9       47.6269       11417.3 -1.000000E+10 -1.000000E+10 -1.000000E+10 -1.000000E+10 -1.000000E+10  0.000000E+00  0.000000E+00  0.000000E+00  0.000000E+00  0.000000E+00  0.000000E+00  0.000000E+00  0.000000E+00  0.000000E+00  0.000000E+00  0.000000E+00  0.000000E+00       Funtag_D
      15  4.715786E+05  4.248152E+05       46763.3       55.2188     0.0109374      0.132638      0.189093      0.241211    0.00396225   -0.00792732    0.00790721       1.00152       Opttag_D
      15       81.2033       1.31532       1.00000  0.000000E+00       1.00000  0.000000E+00      0.941090       93.4270       1.00000       38.9547  0.000000E+00       9.54698  0.000000E+00       9.54698  0.000000E+00  0.000000E+00      -19.0940       83.8070       4.12606        1       Scatag_D
      15       1762.13       5476.54  0.000000E+00  0.000000E+00       2443.82  0.000000E+00       2488.85       10844.2  0.000000E+00       12301.0       47.5577       11399.2 -1.000000E+10 -1.000000E+10 -1.000000E+10 -1.000000E+10 -1.000000E+10  0.000000E+00  0.000000E+00  0.000000E+00  0.000000E+00  0.000000E+00  0.000000E+00  0.000000E+00  0.000000E+00  0.000000E+00  0.000000E+00  0.000000E+00  0.000000E+00       Funtag_D
      16  4.713511E+05  4.248072E+05       46543.9       26.4035     0.0118237      0.146250      0.189133      0.241228    0.00398329   -0.00823196    0.00789641      0.992377       Opttag_D
      16       81.1555       1.30940       1.00000  0.000000E+00       1.00000  0.000000E+00      0.941036       93.2092       1.00000       38.9248  0.000000E+00       9.55908  0.000000E+00       9.55908  0.000000E+00  0.000000E+00      -19.1182       83.8070       4.12606        1       Scatag_D
      16       1757.32       5377.05  0.000000E+00  0.000000E+00       2444.20  0.000000E+00       2493.00       10734.5  0.000000E+00       12303.0       47.5405       11387.3 -1.000000E+10 -1.000000E+10 -1.000000E+10 -1.000000E+10 -1.000000E+10  0.000000E+00  0.000000E+00  0.000000E+00  0.000000E+00  0.000000E+00  0.000000E+00  0.000000E+00  0.000000E+00  0.000000E+00  0.000000E+00  0.000000E+00  0.000000E+00       Funtag_D
      17  4.712270E+05  4.247895E+05       46437.5       20.8100     0.0122327      0.152924      0.189142      0.241224    0.00402969   -0.00833474    0.00789999      0.991983       Opttag_D
      17       81.1374       1.30655       1.00000  0.000000E+00       1.00000  0.000000E+00      0.941013       93.1126       1.00000       38.9112  0.000000E+00       9.56303  0.000000E+00       9.56303  0.000000E+00  0.000000E+00      -19.1261       83.8070       4.12606        1       Scatag_D
      17       1758.92       5372.78  0.000000E+00  0.000000E+00       2444.46  0.000000E+00       2494.09       10636.5  0.000000E+00       12303.6       47.5321       11379.6 -1.000000E+10 -1.000000E+10 -1.000000E+10 -1.000000E+10 -1.000000E+10  0.000000E+00  0.000000E+00  0.000000E+00  0.000000E+00  0.000000E+00  0.000000E+00  0.000000E+00  0.000000E+00  0.000000E+00  0.000000E+00  0.000000E+00  0.000000E+00       Funtag_D
      18  4.710831E+05  4.247597E+05       46323.4       26.8291     0.0130776      0.166382      0.189166      0.241247    0.00410779   -0.00856280    0.00791794      0.993423       Opttag_D
      18       81.1045       1.30123       1.00000  0.000000E+00       1.00000  0.000000E+00      0.940967       92.9248       1.00000       38.8871  0.000000E+00       9.56991  0.000000E+00       9.56991  0.000000E+00  0.000000E+00      -19.1398       83.8070       4.12606        1       Scatag_D
      18       1766.91       5388.39  0.000000E+00  0.000000E+00       2444.98  0.000000E+00       2496.21       10509.5  0.000000E+00       12306.0       47.5788       11363.9 -1.000000E+10 -1.000000E+10 -1.000000E+10 -1.000000E+10 -1.000000E+10  0.000000E+00  0.000000E+00  0.000000E+00  0.000000E+00  0.000000E+00  0.000000E+00  0.000000E+00  0.000000E+00  0.000000E+00  0.000000E+00  0.000000E+00  0.000000E+00       Funtag_D
      19  4.710073E+05  4.247419E+05       46265.4       43.4206     0.0143860      0.186130      0.189215      0.241299    0.00415428   -0.00893109    0.00794790      0.998540       Opttag_D
      19       81.0610       1.29442       1.00000  0.000000E+00       1.00000  0.000000E+00      0.940899       92.6561       1.00000       38.8594  0.000000E+00       9.57914  0.000000E+00       9.57914  0.000000E+00  0.000000E+00      -19.1583       83.8070       4.12606        1       Scatag_D
      19       1780.31       5444.05  0.000000E+00  0.000000E+00       2445.99  0.000000E+00       2499.85       10396.4  0.000000E+00       12309.8       47.7368       11341.4 -1.000000E+10 -1.000000E+10 -1.000000E+10 -1.000000E+10 -1.000000E+10  0.000000E+00  0.000000E+00  0.000000E+00  0.000000E+00  0.000000E+00  0.000000E+00  0.000000E+00  0.000000E+00  0.000000E+00  0.000000E+00  0.000000E+00  0.000000E+00       Funtag_D
      20  4.708773E+05  4.247315E+05       46145.7       21.1542     0.0145045      0.187918      0.189211      0.241305    0.00418137   -0.00897225    0.00793029      0.993104       Opttag_D
      20       81.0612       1.29383       1.00000  0.000000E+00       1.00000  0.000000E+00      0.940894       92.6374       1.00000       38.8580  0.000000E+00       9.57894  0.000000E+00       9.57894  0.000000E+00  0.000000E+00      -19.1579       83.8070       4.12606        1       Scatag_D
      20       1772.43       5384.93  0.000000E+00  0.000000E+00       2446.24  0.000000E+00       2499.36       10347.8  0.000000E+00       12310.4       47.7665       11336.8 -1.000000E+10 -1.000000E+10 -1.000000E+10 -1.000000E+10 -1.000000E+10  0.000000E+00  0.000000E+00  0.000000E+00  0.000000E+00  0.000000E+00  0.000000E+00  0.000000E+00  0.000000E+00  0.000000E+00  0.000000E+00  0.000000E+00  0.000000E+00       Funtag_D
      21  4.707652E+05  4.247190E+05       46046.2       18.0346     0.0150553      0.195641      0.189225      0.241335    0.00421422   -0.00914521    0.00792974      0.993321       Opttag_D
      21       81.0517       1.29142       1.00000  0.000000E+00       1.00000  0.000000E+00      0.940868       92.5403       1.00000       38.8506  0.000000E+00       9.58088  0.000000E+00       9.58088  0.000000E+00  0.000000E+00      -19.1618       83.8070       4.12606        1       Scatag_D
      21       1772.19       5387.28  0.000000E+00  0.000000E+00       2446.94  0.000000E+00       2499.70       10254.7  0.000000E+00       12312.7       47.8279       11324.9 -1.000000E+10 -1.000000E+10 -1.000000E+10 -1.000000E+10 -1.000000E+10  0.000000E+00  0.000000E+00  0.000000E+00  0.000000E+00  0.000000E+00  0.000000E+00  0.000000E+00  0.000000E+00  0.000000E+00  0.000000E+00  0.000000E+00  0.000000E+00       Funtag_D
      22  4.706656E+05  4.247056E+05       45960.0       21.7231     0.0159506      0.207622      0.189251      0.241384    0.00424929   -0.00939295    0.00793300      0.994405       Opttag_D
      22       81.0371       1.28780       1.00000  0.000000E+00       1.00000  0.000000E+00      0.940828       92.3862       1.00000       38.8406  0.000000E+00       9.58371  0.000000E+00       9.58371  0.000000E+00  0.000000E+00      -19.1674       83.8070       4.12606        1       Scatag_D
      22       1773.64       5399.05  0.000000E+00  0.000000E+00       2448.17  0.000000E+00       2500.76       10167.1  0.000000E+00       12316.5       47.8705       11306.9 -1.000000E+10 -1.000000E+10 -1.000000E+10 -1.000000E+10 -1.000000E+10  0.000000E+00  0.000000E+00  0.000000E+00  0.000000E+00  0.000000E+00  0.000000E+00  0.000000E+00  0.000000E+00  0.000000E+00  0.000000E+00  0.000000E+00  0.000000E+00       Funtag_D
      23  4.706011E+05  4.246816E+05       45919.5       51.9929     0.0181021      0.247031      0.189311      0.241506    0.00431211   -0.00991935    0.00797417       1.00438       Opttag_D
      23       81.0157       1.28046       1.00000  0.000000E+00       1.00000  0.000000E+00      0.940743       92.0484       1.00000       38.8270  0.000000E+00       9.58688  0.000000E+00       9.58688  0.000000E+00  0.000000E+00      -19.1738       83.8070       4.12606        1       Scatag_D
      23       1792.10       5507.93  0.000000E+00  0.000000E+00       2451.46  0.000000E+00       2503.46       10024.4  0.000000E+00       12326.5       47.8202       11265.8 -1.000000E+10 -1.000000E+10 -1.000000E+10 -1.000000E+10 -1.000000E+10  0.000000E+00  0.000000E+00  0.000000E+00  0.000000E+00  0.000000E+00  0.000000E+00  0.000000E+00  0.000000E+00  0.000000E+00  0.000000E+00  0.000000E+00  0.000000E+00       Funtag_D
      24  4.704430E+05  4.246770E+05       45766.0       20.3821     0.0186867      0.258175      0.189329      0.241537    0.00432412    -0.0100546    0.00794179      0.995493       Opttag_D
      24       81.0126       1.27871       1.00000  0.000000E+00       1.00000  0.000000E+00      0.940720       91.9624       1.00000       38.8258  0.000000E+00       9.58715  0.000000E+00       9.58715  0.000000E+00  0.000000E+00      -19.1743       83.8070       4.12606        1       Scatag_D
      24       1777.58       5410.87  0.000000E+00  0.000000E+00       2452.70  0.000000E+00       2504.37       9990.13  0.000000E+00       12330.2       47.7973       11252.3 -1.000000E+10 -1.000000E+10 -1.000000E+10 -1.000000E+10 -1.000000E+10  0.000000E+00  0.000000E+00  0.000000E+00  0.000000E+00  0.000000E+00  0.000000E+00  0.000000E+00  0.000000E+00  0.000000E+00  0.000000E+00  0.000000E+00  0.000000E+00       Funtag_D
      25  4.703723E+05  4.246598E+05       45712.5       14.4973     0.0190468      0.265757      0.189330      0.241542    0.00436927    -0.0101264    0.00793577      0.995190       Opttag_D
      25       81.0160       1.27761       1.00000  0.000000E+00       1.00000  0.000000E+00      0.940709       91.9184       1.00000       38.8260  0.000000E+00       9.58609  0.000000E+00       9.58609  0.000000E+00  0.000000E+00      -19.1722       83.8070       4.12606        1       Scatag_D
      25       1774.88       5407.58  0.000000E+00  0.000000E+00       2453.66  0.000000E+00       2504.47       9947.95  0.000000E+00       12332.7       47.7369       11243.6 -1.000000E+10 -1.000000E+10 -1.000000E+10 -1.000000E+10 -1.000000E+10  0.000000E+00  0.000000E+00  0.000000E+00  0.000000E+00  0.000000E+00  0.000000E+00  0.000000E+00  0.000000E+00  0.000000E+00  0.000000E+00  0.000000E+00  0.000000E+00       Funtag_D
      26  4.702857E+05  4.246290E+05       45656.7       19.8870     0.0200254      0.294103      0.189336      0.241573    0.00445005    -0.0103342    0.00793543      0.996401       Opttag_D
      26       81.0234       1.27501       1.00000  0.000000E+00       1.00000  0.000000E+00      0.940677       91.8008       1.00000       38.8271  0.000000E+00       9.58367  0.000000E+00       9.58367  0.000000E+00  0.000000E+00      -19.1673       83.8070       4.12606        1       Scatag_D
      26       1774.73       5420.75  0.000000E+00  0.000000E+00       2456.04  0.000000E+00       2505.38       9890.12  0.000000E+00       12339.3       47.6739       11222.7 -1.000000E+10 -1.000000E+10 -1.000000E+10 -1.000000E+10 -1.000000E+10  0.000000E+00  0.000000E+00  0.000000E+00  0.000000E+00  0.000000E+00  0.000000E+00  0.000000E+00  0.000000E+00  0.000000E+00  0.000000E+00  0.000000E+00  0.000000E+00       Funtag_D
      27  4.702109E+05  4.245816E+05       45629.2       36.3911     0.0216470      0.342766      0.189346      0.241625    0.00457407    -0.0106843    0.00794588       1.00064       Opttag_D
      27       81.0439       1.27156       1.00000  0.000000E+00       1.00000  0.000000E+00      0.940625       91.6322       1.00000       38.8335  0.000000E+00       9.57818  0.000000E+00       9.57818  0.000000E+00  0.000000E+00      -19.1564       83.8070       4.12606        1       Scatag_D
      27       1779.41       5466.96  0.000000E+00  0.000000E+00       2460.19  0.000000E+00       2507.52       9821.69  0.000000E+00       12351.8       47.6935       11194.0 -1.000000E+10 -1.000000E+10 -1.000000E+10 -1.000000E+10 -1.000000E+10  0.000000E+00  0.000000E+00  0.000000E+00  0.000000E+00  0.000000E+00  0.000000E+00  0.000000E+00  0.000000E+00  0.000000E+00  0.000000E+00  0.000000E+00  0.000000E+00       Funtag_D
      28  4.701120E+05  4.245607E+05       45551.3       19.2126     0.0224495      0.366615      0.189347      0.241647    0.00462892    -0.0108446    0.00793510      0.997082       Opttag_D
      28       81.0575       1.27024       1.00000  0.000000E+00       1.00000  0.000000E+00      0.940603       91.5586       1.00000       38.8377  0.000000E+00       9.57498  0.000000E+00       9.57498  0.000000E+00  0.000000E+00      -19.1500       83.8070       4.12606        1       Scatag_D
      28       1774.58       5428.16  0.000000E+00  0.000000E+00       2462.22  0.000000E+00       2508.72       9789.09  0.000000E+00       12358.0       47.7332       11182.8 -1.000000E+10 -1.000000E+10 -1.000000E+10 -1.000000E+10 -1.000000E+10  0.000000E+00  0.000000E+00  0.000000E+00  0.000000E+00  0.000000E+00  0.000000E+00  0.000000E+00  0.000000E+00  0.000000E+00  0.000000E+00  0.000000E+00  0.000000E+00       Funtag_D
      29  4.700354E+05  4.245350E+05       45500.5       14.1758     0.0231213      0.386717      0.189338      0.241659    0.00469616    -0.0109808    0.00793520      0.996688       Opttag_D
      29       81.0756       1.26950       1.00000  0.000000E+00       1.00000  0.000000E+00      0.940587       91.5149       1.00000       38.8419  0.000000E+00       9.57123  0.000000E+00       9.57123  0.000000E+00  0.000000E+00      -19.1425       83.8070       4.12606        1       Scatag_D
      29       1774.63       5423.87  0.000000E+00  0.000000E+00       2464.04  0.000000E+00       2509.81       9741.92  0.000000E+00       12363.8       47.8042       11174.6 -1.000000E+10 -1.000000E+10 -1.000000E+10 -1.000000E+10 -1.000000E+10  0.000000E+00  0.000000E+00  0.000000E+00  0.000000E+00  0.000000E+00  0.000000E+00  0.000000E+00  0.000000E+00  0.000000E+00  0.000000E+00  0.000000E+00  0.000000E+00       Funtag_D
      30  4.699660E+05  4.244989E+05       45467.1       17.8771     0.0240239      0.411718      0.189324      0.241673    0.00479069    -0.0111415    0.00793799      0.997339       Opttag_D
      30       81.1030       1.26875       1.00000  0.000000E+00       1.00000  0.000000E+00      0.940567       91.4645       1.00000       38.8487  0.000000E+00       9.56590  0.000000E+00       9.56590  0.000000E+00  0.000000E+00      -19.1318       83.8070       4.12606        1       Scatag_D
      30       1775.88       5430.96  0.000000E+00  0.000000E+00       2466.33  0.000000E+00       2511.42       9696.95  0.000000E+00       12372.0       47.8654       11165.6 -1.000000E+10 -1.000000E+10 -1.000000E+10 -1.000000E+10 -1.000000E+10  0.000000E+00  0.000000E+00  0.000000E+00  0.000000E+00  0.000000E+00  0.000000E+00  0.000000E+00  0.000000E+00  0.000000E+00  0.000000E+00  0.000000E+00  0.000000E+00       Funtag_D
      31  4.699039E+05  4.244351E+05       45468.8       31.0390     0.0257738      0.454333      0.189293      0.241700    0.00495774    -0.0114174    0.00794818       1.00029       Opttag_D
      31       81.1594       1.26784       1.00000  0.000000E+00       1.00000  0.000000E+00      0.940537       91.3777       1.00000       38.8648  0.000000E+00       9.55529  0.000000E+00       9.55529  0.000000E+00  0.000000E+00      -19.1106       83.8070       4.12606        1       Scatag_D
      31       1780.44       5463.12  0.000000E+00  0.000000E+00       2470.89  0.000000E+00       2515.28       9648.58  0.000000E+00       12389.6       47.9148       11153.0 -1.000000E+10 -1.000000E+10 -1.000000E+10 -1.000000E+10 -1.000000E+10  0.000000E+00  0.000000E+00  0.000000E+00  0.000000E+00  0.000000E+00  0.000000E+00  0.000000E+00  0.000000E+00  0.000000E+00  0.000000E+00  0.000000E+00  0.000000E+00       Funtag_D
      32  4.698186E+05  4.244079E+05       45410.7       16.8981     0.0263214      0.464170      0.189276      0.241697    0.00502892    -0.0114774    0.00793915      0.997399       Opttag_D
      32       81.1813       1.26776       1.00000  0.000000E+00       1.00000  0.000000E+00      0.940531       91.3609       1.00000       38.8704  0.000000E+00       9.55153  0.000000E+00       9.55153  0.000000E+00  0.000000E+00      -19.1031       83.8070       4.12606        1       Scatag_D
      32       1776.40       5431.61  0.000000E+00  0.000000E+00       2471.83  0.000000E+00       2516.50       9624.73  0.000000E+00       12395.5       47.8956       11146.2 -1.000000E+10 -1.000000E+10 -1.000000E+10 -1.000000E+10 -1.000000E+10  0.000000E+00  0.000000E+00  0.000000E+00  0.000000E+00  0.000000E+00  0.000000E+00  0.000000E+00  0.000000E+00  0.000000E+00  0.000000E+00  0.000000E+00  0.000000E+00       Funtag_D
      33  4.697480E+05  4.243717E+05       45376.3       14.6955     0.0269385      0.471439      0.189251      0.241681    0.00512383    -0.0115221    0.00793973      0.997520       Opttag_D
      33       81.2087       1.26776       1.00000  0.000000E+00       1.00000  0.000000E+00      0.940529       91.3463       1.00000       38.8767  0.000000E+00       9.54720  0.000000E+00       9.54720  0.000000E+00  0.000000E+00      -19.0944       83.8070       4.12606        1       Scatag_D
      33       1776.66       5432.93  0.000000E+00  0.000000E+00       2472.93  0.000000E+00       2517.83       9589.31  0.000000E+00       12402.5       47.8497       11136.3 -1.000000E+10 -1.000000E+10 -1.000000E+10 -1.000000E+10 -1.000000E+10  0.000000E+00  0.000000E+00  0.000000E+00  0.000000E+00  0.000000E+00  0.000000E+00  0.000000E+00  0.000000E+00  0.000000E+00  0.000000E+00  0.000000E+00  0.000000E+00       Funtag_D
      34  4.696722E+05  4.243161E+05       45356.1       17.0645     0.0280143      0.483576      0.189214      0.241658    0.00526936    -0.0116084    0.00794256      0.998163       Opttag_D
      34       81.2544       1.26782       1.00000  0.000000E+00       1.00000  0.000000E+00      0.940530       91.3174       1.00000       38.8883  0.000000E+00       9.53997  0.000000E+00       9.53997  0.000000E+00  0.000000E+00      -19.0799       83.8070       4.12606        1       Scatag_D
      34       1777.92       5439.94  0.000000E+00  0.000000E+00       2474.83  0.000000E+00       2520.37       9559.38  0.000000E+00       12415.1       47.8454       11120.7 -1.000000E+10 -1.000000E+10 -1.000000E+10 -1.000000E+10 -1.000000E+10  0.000000E+00  0.000000E+00  0.000000E+00  0.000000E+00  0.000000E+00  0.000000E+00  0.000000E+00  0.000000E+00  0.000000E+00  0.000000E+00  0.000000E+00  0.000000E+00       Funtag_D
      35  4.696116E+05  4.242047E+05       45406.8       38.2596     0.0304292      0.533469      0.189130      0.241622    0.00556110    -0.0117707    0.00797795       1.00392       Opttag_D
      35       81.3568       1.26851       1.00000  0.000000E+00       1.00000  0.000000E+00      0.940540       91.2598       1.00000       38.9150  0.000000E+00       9.52419  0.000000E+00       9.52419  0.000000E+00  0.000000E+00      -19.0484       83.8070       4.12606        1       Scatag_D
      35       1793.80       5502.82  0.000000E+00  0.000000E+00       2478.73  0.000000E+00       2526.49       9524.89  0.000000E+00       12444.3       47.9871       11087.8 -1.000000E+10 -1.000000E+10 -1.000000E+10 -1.000000E+10 -1.000000E+10  0.000000E+00  0.000000E+00  0.000000E+00  0.000000E+00  0.000000E+00  0.000000E+00  0.000000E+00  0.000000E+00  0.000000E+00  0.000000E+00  0.000000E+00  0.000000E+00       Funtag_D
      36  4.694983E+05  4.241720E+05       45326.2       16.9186     0.0312673      0.555051      0.189105      0.241611    0.00564669    -0.0118277    0.00795054      0.998858       Opttag_D
      36       81.3915       1.26886       1.00000  0.000000E+00       1.00000  0.000000E+00      0.940548       91.2392       1.00000       38.9251  0.000000E+00       9.51893  0.000000E+00       9.51893  0.000000E+00  0.000000E+00      -19.0379       83.8070       4.12606        1       Scatag_D
      36       1781.50       5447.51  0.000000E+00  0.000000E+00       2480.02  0.000000E+00       2528.70       9510.02  0.000000E+00       12454.8       48.0724       11075.6 -1.000000E+10 -1.000000E+10 -1.000000E+10 -1.000000E+10 -1.000000E+10  0.000000E+00  0.000000E+00  0.000000E+00  0.000000E+00  0.000000E+00  0.000000E+00  0.000000E+00  0.000000E+00  0.000000E+00  0.000000E+00  0.000000E+00  0.000000E+00       Funtag_D
      37  4.694324E+05  4.241405E+05       45292.0       12.9945     0.0319530      0.573924      0.189080      0.241598    0.00572940    -0.0118585    0.00794749      0.998846       Opttag_D
      37       81.4210       1.26916       1.00000  0.000000E+00       1.00000  0.000000E+00      0.940559       91.2246       1.00000       38.9310  0.000000E+00       9.51478  0.000000E+00       9.51478  0.000000E+00  0.000000E+00      -19.0296       83.8070       4.12606        1       Scatag_D
      37       1780.13       5447.39  0.000000E+00  0.000000E+00       2480.94  0.000000E+00       2530.14       9477.59  0.000000E+00       12463.3       48.1911       11064.3 -1.000000E+10 -1.000000E+10 -1.000000E+10 -1.000000E+10 -1.000000E+10  0.000000E+00  0.000000E+00  0.000000E+00  0.000000E+00  0.000000E+00  0.000000E+00  0.000000E+00  0.000000E+00  0.000000E+00  0.000000E+00  0.000000E+00  0.000000E+00       Funtag_D
      38  4.693572E+05  4.240852E+05       45272.1       15.8443     0.0332999      0.610861      0.189033      0.241574    0.00587417    -0.0118945    0.00795156      0.999571       Opttag_D
      38       81.4781       1.26994       1.00000  0.000000E+00       1.00000  0.000000E+00      0.940578       91.1985       1.00000       38.9426  0.000000E+00       9.50677  0.000000E+00       9.50677  0.000000E+00  0.000000E+00      -19.0135       83.8070       4.12606        1       Scatag_D
      38       1781.96       5455.29  0.000000E+00  0.000000E+00       2482.71  0.000000E+00       2533.01       9446.48  0.000000E+00       12480.2       48.3647       11044.0 -1.000000E+10 -1.000000E+10 -1.000000E+10 -1.000000E+10 -1.000000E+10  0.000000E+00  0.000000E+00  0.000000E+00  0.000000E+00  0.000000E+00  0.000000E+00  0.000000E+00  0.000000E+00  0.000000E+00  0.000000E+00  0.000000E+00  0.000000E+00       Funtag_D
      39  4.692666E+05  4.239982E+05       45268.4       28.7134     0.0357697      0.680905      0.188954      0.241540    0.00610193    -0.0118771    0.00797251       1.00281       Opttag_D
      39       81.5797       1.27167       1.00000  0.000000E+00       1.00000  0.000000E+00      0.940614       91.1532       1.00000       38.9627  0.000000E+00       9.49284  0.000000E+00       9.49284  0.000000E+00  0.000000E+00      -18.9857       83.8070       4.12606        1       Scatag_D
      39       1791.36       5490.70  0.000000E+00  0.000000E+00       2485.72  0.000000E+00       2537.69       9395.98  0.000000E+00       12512.0       48.5525       11006.4 -1.000000E+10 -1.000000E+10 -1.000000E+10 -1.000000E+10 -1.000000E+10  0.000000E+00  0.000000E+00  0.000000E+00  0.000000E+00  0.000000E+00  0.000000E+00  0.000000E+00  0.000000E+00  0.000000E+00  0.000000E+00  0.000000E+00  0.000000E+00       Funtag_D
      40  4.691631E+05  4.239311E+05       45232.0       17.9099     0.0382581      0.751016      0.188893      0.241511    0.00627772    -0.0118306    0.00798170       1.00048       Opttag_D
      40       81.6788       1.27382       1.00000  0.000000E+00       1.00000  0.000000E+00      0.940647       91.1086       1.00000       38.9864  0.000000E+00       9.47915  0.000000E+00       9.47915  0.000000E+00  0.000000E+00      -18.9583       83.8070       4.12606        1       Scatag_D
      40       1795.49       5465.25  0.000000E+00  0.000000E+00       2488.74  0.000000E+00       2542.91       9379.55  0.000000E+00       12545.2       48.7428       10966.2 -1.000000E+10 -1.000000E+10 -1.000000E+10 -1.000000E+10 -1.000000E+10  0.000000E+00  0.000000E+00  0.000000E+00  0.000000E+00  0.000000E+00  0.000000E+00  0.000000E+00  0.000000E+00  0.000000E+00  0.000000E+00  0.000000E+00  0.000000E+00       Funtag_D
      41  4.690863E+05  4.238996E+05       45186.8       14.1241     0.0393832      0.787701      0.188863      0.241492    0.00636024    -0.0117473    0.00798813      0.999958       Opttag_D
      41       81.7216       1.27467       1.00000  0.000000E+00       1.00000  0.000000E+00      0.940665       91.0862       1.00000       38.9947  0.000000E+00       9.47357  0.000000E+00       9.47357  0.000000E+00  0.000000E+00      -18.9471       83.8070       4.12606        1       Scatag_D
      41       1798.38       5459.52  0.000000E+00  0.000000E+00       2490.22  0.000000E+00       2544.52       9344.26  0.000000E+00       12560.3       48.7851       10940.8 -1.000000E+10 -1.000000E+10 -1.000000E+10 -1.000000E+10 -1.000000E+10  0.000000E+00  0.000000E+00  0.000000E+00  0.000000E+00  0.000000E+00  0.000000E+00  0.000000E+00  0.000000E+00  0.000000E+00  0.000000E+00  0.000000E+00  0.000000E+00       Funtag_D
      42  4.689836E+05  4.238431E+05       45140.6       19.9607     0.0414216      0.859711      0.188813      0.241443    0.00650827    -0.0115422    0.00799991       1.00071       Opttag_D
      42       81.7960       1.27599       1.00000  0.000000E+00       1.00000  0.000000E+00      0.940702       91.0389       1.00000       39.0091  0.000000E+00       9.46416  0.000000E+00       9.46416  0.000000E+00  0.000000E+00      -18.9283       83.8070       4.12606        1       Scatag_D
      42       1803.69       5467.77  0.000000E+00  0.000000E+00       2492.46  0.000000E+00       2546.62       9297.10  0.000000E+00       12587.5       48.8798       10896.5 -1.000000E+10 -1.000000E+10 -1.000000E+10 -1.000000E+10 -1.000000E+10  0.000000E+00  0.000000E+00  0.000000E+00  0.000000E+00  0.000000E+00  0.000000E+00  0.000000E+00  0.000000E+00  0.000000E+00  0.000000E+00  0.000000E+00  0.000000E+00       Funtag_D
      43  4.689325E+05  4.238032E+05       45129.4       26.2749     0.0426522      0.906286      0.188782      0.241411    0.00661279    -0.0114110    0.00800514       1.00152       Opttag_D
      43       81.8376       1.27625       1.00000  0.000000E+00       1.00000  0.000000E+00      0.940738       90.9940       1.00000       39.0175  0.000000E+00       9.45943  0.000000E+00       9.45943  0.000000E+00  0.000000E+00      -18.9189       83.8070       4.12606        1       Scatag_D
      43       1806.05       5476.59  0.000000E+00  0.000000E+00       2493.88  0.000000E+00       2547.14       9273.04  0.000000E+00       12603.4       49.0077       10880.3 -1.000000E+10 -1.000000E+10 -1.000000E+10 -1.000000E+10 -1.000000E+10  0.000000E+00  0.000000E+00  0.000000E+00  0.000000E+00  0.000000E+00  0.000000E+00  0.000000E+00  0.000000E+00  0.000000E+00  0.000000E+00  0.000000E+00  0.000000E+00       Funtag_D
      44  4.688609E+05  4.238089E+05       45052.0       13.6015     0.0418346      0.884899      0.188795      0.241412    0.00659780    -0.0114114    0.00798198      0.999618       Opttag_D
      44       81.8052       1.27500       1.00000  0.000000E+00       1.00000  0.000000E+00      0.940733       90.9987       1.00000       39.0063  0.000000E+00       9.46405  0.000000E+00       9.46405  0.000000E+00  0.000000E+00      -18.9281       83.8070       4.12606        1       Scatag_D
      44       1795.61       5455.80  0.000000E+00  0.000000E+00       2492.99  0.000000E+00       2544.56       9247.94  0.000000E+00       12591.0       48.9772       10875.2 -1.000000E+10 -1.000000E+10 -1.000000E+10 -1.000000E+10 -1.000000E+10  0.000000E+00  0.000000E+00  0.000000E+00  0.000000E+00  0.000000E+00  0.000000E+00  0.000000E+00  0.000000E+00  0.000000E+00  0.000000E+00  0.000000E+00  0.000000E+00       Funtag_D
      45  4.688024E+05  4.237902E+05       45012.2       10.2309     0.0422542      0.898920      0.188783      0.241393    0.00664660    -0.0113858    0.00797459      0.999470       Opttag_D
      45       81.8180       1.27471       1.00000  0.000000E+00       1.00000  0.000000E+00      0.940751       90.9699       1.00000       39.0104  0.000000E+00       9.46274  0.000000E+00       9.46274  0.000000E+00  0.000000E+00      -18.9255       83.8070       4.12606        1       Scatag_D
      45       1792.29       5454.19  0.000000E+00  0.000000E+00       2493.16  0.000000E+00       2544.07       9214.75  0.000000E+00       12595.1       49.0797       10869.5 -1.000000E+10 -1.000000E+10 -1.000000E+10 -1.000000E+10 -1.000000E+10  0.000000E+00  0.000000E+00  0.000000E+00  0.000000E+00  0.000000E+00  0.000000E+00  0.000000E+00  0.000000E+00  0.000000E+00  0.000000E+00  0.000000E+00  0.000000E+00       Funtag_D
      46  4.687521E+05  4.237625E+05       44989.6       12.4793     0.0430114      0.919451      0.188765      0.241364    0.00671913    -0.0113429    0.00797369      0.999597       Opttag_D
      46       81.8427       1.27449       1.00000  0.000000E+00       1.00000  0.000000E+00      0.940778       90.9244       1.00000       39.0185  0.000000E+00       9.46007  0.000000E+00       9.46007  0.000000E+00  0.000000E+00      -18.9201       83.8070       4.12606        1       Scatag_D
      46       1791.89       5455.58  0.000000E+00  0.000000E+00       2493.64  0.000000E+00       2543.86       9185.13  0.000000E+00       12603.0       49.2122       10867.3 -1.000000E+10 -1.000000E+10 -1.000000E+10 -1.000000E+10 -1.000000E+10  0.000000E+00  0.000000E+00  0.000000E+00  0.000000E+00  0.000000E+00  0.000000E+00  0.000000E+00  0.000000E+00  0.000000E+00  0.000000E+00  0.000000E+00  0.000000E+00       Funtag_D
      47  4.687137E+05  4.237193E+05       44994.4       25.6974     0.0441498      0.936037      0.188739      0.241311    0.00683248    -0.0112471    0.00798259       1.00113       Opttag_D
      47       81.8800       1.27400       1.00000  0.000000E+00       1.00000  0.000000E+00      0.940823       90.8413       1.00000       39.0306  0.000000E+00       9.45632  0.000000E+00       9.45632  0.000000E+00  0.000000E+00      -18.9126       83.8070       4.12606        1       Scatag_D
      47       1795.89       5472.33  0.000000E+00  0.000000E+00       2494.07  0.000000E+00       2543.45       9156.13  0.000000E+00       12612.4       49.3291       10870.7 -1.000000E+10 -1.000000E+10 -1.000000E+10 -1.000000E+10 -1.000000E+10  0.000000E+00  0.000000E+00  0.000000E+00  0.000000E+00  0.000000E+00  0.000000E+00  0.000000E+00  0.000000E+00  0.000000E+00  0.000000E+00  0.000000E+00  0.000000E+00       Funtag_D
      48  4.686484E+05  4.237047E+05       44943.7       11.6176     0.0445932      0.939325      0.188729      0.241292    0.00687062    -0.0111928    0.00797745      0.999281       Opttag_D
      48       81.8953       1.27397       1.00000  0.000000E+00       1.00000  0.000000E+00      0.940835       90.8143       1.00000       39.0350  0.000000E+00       9.45466  0.000000E+00       9.45466  0.000000E+00  0.000000E+00      -18.9093       83.8070       4.12606        1       Scatag_D
      48       1793.58       5452.13  0.000000E+00  0.000000E+00       2494.36  0.000000E+00       2543.56       9123.04  0.000000E+00       12616.0       49.3362       10871.7 -1.000000E+10 -1.000000E+10 -1.000000E+10 -1.000000E+10 -1.000000E+10  0.000000E+00  0.000000E+00  0.000000E+00  0.000000E+00  0.000000E+00  0.000000E+00  0.000000E+00  0.000000E+00  0.000000E+00  0.000000E+00  0.000000E+00  0.000000E+00       Funtag_D
      49  4.685993E+05  4.236871E+05       44912.2       10.4095     0.0450425      0.932905      0.188716      0.241264    0.00691682    -0.0111008    0.00797923      0.999048       Opttag_D
      49       81.9119       1.27388       1.00000  0.000000E+00       1.00000  0.000000E+00      0.940848       90.7825       1.00000       39.0391  0.000000E+00       9.45295  0.000000E+00       9.45295  0.000000E+00  0.000000E+00      -18.9059       83.8070       4.12606        1       Scatag_D
      49       1794.38       5449.59  0.000000E+00  0.000000E+00       2494.65  0.000000E+00       2543.61       9093.43  0.000000E+00       12618.3       49.2888       10869.0 -1.000000E+10 -1.000000E+10 -1.000000E+10 -1.000000E+10 -1.000000E+10  0.000000E+00  0.000000E+00  0.000000E+00  0.000000E+00  0.000000E+00  0.000000E+00  0.000000E+00  0.000000E+00  0.000000E+00  0.000000E+00  0.000000E+00  0.000000E+00       Funtag_D
      50  4.685483E+05  4.236612E+05       44887.0       13.2309     0.0457165      0.919384      0.188698      0.241219    0.00698445    -0.0109401    0.00798163      0.999040       Opttag_D
      50       81.9368       1.27378       1.00000  0.000000E+00       1.00000  0.000000E+00      0.940867       90.7345       1.00000       39.0457  0.000000E+00       9.45031  0.000000E+00       9.45031  0.000000E+00  0.000000E+00      -18.9006       83.8070       4.12606        1       Scatag_D
      50       1795.45       5449.50  0.000000E+00  0.000000E+00       2494.98  0.000000E+00       2544.04       9067.42  0.000000E+00       12621.3       49.2579       10865.1 -1.000000E+10 -1.000000E+10 -1.000000E+10 -1.000000E+10 -1.000000E+10  0.000000E+00  0.000000E+00  0.000000E+00  0.000000E+00  0.000000E+00  0.000000E+00  0.000000E+00  0.000000E+00  0.000000E+00  0.000000E+00  0.000000E+00  0.000000E+00       Funtag_D
      51  4.684915E+05  4.236095E+05       44882.0       27.5778     0.0472444      0.882473      0.188658      0.241115    0.00711990    -0.0105366    0.00799268       1.00038       Opttag_D
      51       81.9934       1.27386       1.00000  0.000000E+00       1.00000  0.000000E+00      0.940908       90.6344       1.00000       39.0627  0.000000E+00       9.44431  0.000000E+00       9.44431  0.000000E+00  0.000000E+00      -18.8886       83.8070       4.12606        1       Scatag_D
      51       1800.43       5464.15  0.000000E+00  0.000000E+00       2495.69  0.000000E+00       2546.41       9038.27  0.000000E+00       12628.9       49.3382       10858.8 -1.000000E+10 -1.000000E+10 -1.000000E+10 -1.000000E+10 -1.000000E+10  0.000000E+00  0.000000E+00  0.000000E+00  0.000000E+00  0.000000E+00  0.000000E+00  0.000000E+00  0.000000E+00  0.000000E+00  0.000000E+00  0.000000E+00  0.000000E+00       Funtag_D
      52  4.684140E+05  4.235869E+05       44827.1       13.8962     0.0480979      0.860501      0.188640      0.241061    0.00717917    -0.0103198    0.00798854      0.999234       Opttag_D
      52       82.0246       1.27413       1.00000  0.000000E+00       1.00000  0.000000E+00      0.940929       90.5830       1.00000       39.0732  0.000000E+00       9.44079  0.000000E+00       9.44079  0.000000E+00  0.000000E+00      -18.8816       83.8070       4.12606        1       Scatag_D
      52       1798.57       5451.62  0.000000E+00  0.000000E+00       2496.11  0.000000E+00       2548.27       8990.69  0.000000E+00       12633.6       49.3944       10858.8 -1.000000E+10 -1.000000E+10 -1.000000E+10 -1.000000E+10 -1.000000E+10  0.000000E+00  0.000000E+00  0.000000E+00  0.000000E+00  0.000000E+00  0.000000E+00  0.000000E+00  0.000000E+00  0.000000E+00  0.000000E+00  0.000000E+00  0.000000E+00       Funtag_D
      53  4.683684E+05  4.235720E+05       44796.4       9.24533     0.0484820      0.845826      0.188626      0.241038    0.00721807    -0.0102053    0.00798495      0.999037       Opttag_D
      53       82.0397       1.27427       1.00000  0.000000E+00       1.00000  0.000000E+00      0.940936       90.5635       1.00000       39.0771  0.000000E+00       9.43917  0.000000E+00       9.43917  0.000000E+00  0.000000E+00      -18.8783       83.8070       4.12606        1       Scatag_D
      53       1796.95       5449.47  0.000000E+00  0.000000E+00       2496.31  0.000000E+00       2549.19       8963.68  0.000000E+00       12635.0       49.4724       10856.3 -1.000000E+10 -1.000000E+10 -1.000000E+10 -1.000000E+10 -1.000000E+10  0.000000E+00  0.000000E+00  0.000000E+00  0.000000E+00  0.000000E+00  0.000000E+00  0.000000E+00  0.000000E+00  0.000000E+00  0.000000E+00  0.000000E+00  0.000000E+00       Funtag_D
      54  4.683259E+05  4.235485E+05       44777.5       11.3359     0.0491230      0.823517      0.188603      0.241001    0.00727984    -0.0100397    0.00798443      0.999249       Opttag_D
      54       82.0650       1.27454       1.00000  0.000000E+00       1.00000  0.000000E+00      0.940949       90.5332       1.00000       39.0838  0.000000E+00       9.43684  0.000000E+00       9.43684  0.000000E+00  0.000000E+00      -18.8737       83.8070       4.12606        1       Scatag_D
      54       1796.72       5451.78  0.000000E+00  0.000000E+00       2496.70  0.000000E+00       2550.68       8939.96  0.000000E+00       12637.8       49.5712       10854.3 -1.000000E+10 -1.000000E+10 -1.000000E+10 -1.000000E+10 -1.000000E+10  0.000000E+00  0.000000E+00  0.000000E+00  0.000000E+00  0.000000E+00  0.000000E+00  0.000000E+00  0.000000E+00  0.000000E+00  0.000000E+00  0.000000E+00  0.000000E+00       Funtag_D
      55  4.682911E+05  4.235167E+05       44774.4       18.0437     0.0502157      0.792693      0.188573      0.240956    0.00736303   -0.00983096    0.00798544      0.999536       Opttag_D
      55       82.1065       1.27521       1.00000  0.000000E+00       1.00000  0.000000E+00      0.940973       90.4827       1.00000       39.0956  0.000000E+00       9.43321  0.000000E+00       9.43321  0.000000E+00  0.000000E+00      -18.8664       83.8070       4.12606        1       Scatag_D
      55       1797.17       5454.91  0.000000E+00  0.000000E+00       2497.41  0.000000E+00       2553.12       8923.28  0.000000E+00       12643.3       49.6325       10855.6 -1.000000E+10 -1.000000E+10 -1.000000E+10 -1.000000E+10 -1.000000E+10  0.000000E+00  0.000000E+00  0.000000E+00  0.000000E+00  0.000000E+00  0.000000E+00  0.000000E+00  0.000000E+00  0.000000E+00  0.000000E+00  0.000000E+00  0.000000E+00       Funtag_D
      56  4.682477E+05  4.235036E+05       44744.0       10.4281     0.0506143      0.784095      0.188559      0.240940    0.00739723   -0.00977383    0.00798278      0.999337       Opttag_D
      56       82.1222       1.27546       1.00000  0.000000E+00       1.00000  0.000000E+00      0.940983       90.4660       1.00000       39.1000  0.000000E+00       9.43220  0.000000E+00       9.43220  0.000000E+00  0.000000E+00      -18.8644       83.8070       4.12606        1       Scatag_D
      56       1795.97       5452.74  0.000000E+00  0.000000E+00       2497.75  0.000000E+00       2553.73       8894.00  0.000000E+00       12645.3       49.6013       10854.9 -1.000000E+10 -1.000000E+10 -1.000000E+10 -1.000000E+10 -1.000000E+10  0.000000E+00  0.000000E+00  0.000000E+00  0.000000E+00  0.000000E+00  0.000000E+00  0.000000E+00  0.000000E+00  0.000000E+00  0.000000E+00  0.000000E+00  0.000000E+00       Funtag_D
      57  4.682041E+05  4.234853E+05       44718.8       9.51356     0.0512722      0.774379      0.188542      0.240923    0.00744526   -0.00971322    0.00797937      0.999294       Opttag_D
      57       82.1476       1.27593       1.00000  0.000000E+00       1.00000  0.000000E+00      0.940999       90.4386       1.00000       39.1075  0.000000E+00       9.43053  0.000000E+00       9.43053  0.000000E+00  0.000000E+00      -18.8611       83.8070       4.12606        1       Scatag_D
      57       1794.44       5452.27  0.000000E+00  0.000000E+00       2498.36  0.000000E+00       2554.40       8867.71  0.000000E+00       12648.7       49.4979       10853.4 -1.000000E+10 -1.000000E+10 -1.000000E+10 -1.000000E+10 -1.000000E+10  0.000000E+00  0.000000E+00  0.000000E+00  0.000000E+00  0.000000E+00  0.000000E+00  0.000000E+00  0.000000E+00  0.000000E+00  0.000000E+00  0.000000E+00  0.000000E+00       Funtag_D
      58  4.681644E+05  4.234636E+05       44700.8       11.2647     0.0521223      0.764282      0.188524      0.240902    0.00750203   -0.00963675    0.00797754      0.999331       Opttag_D
      58       82.1798       1.27656       1.00000  0.000000E+00       1.00000  0.000000E+00      0.941021       90.4035       1.00000       39.1183  0.000000E+00       9.42802  0.000000E+00       9.42802  0.000000E+00  0.000000E+00      -18.8560       83.8070       4.12606        1       Scatag_D
      58       1793.62       5452.68  0.000000E+00  0.000000E+00       2499.13  0.000000E+00       2555.13       8844.70  0.000000E+00       12653.1       49.3887       10853.0 -1.000000E+10 -1.000000E+10 -1.000000E+10 -1.000000E+10 -1.000000E+10  0.000000E+00  0.000000E+00  0.000000E+00  0.000000E+00  0.000000E+00  0.000000E+00  0.000000E+00  0.000000E+00  0.000000E+00  0.000000E+00  0.000000E+00  0.000000E+00       Funtag_D
      59  4.681260E+05  4.234191E+05       44707.0       24.0262     0.0543354      0.762207      0.188484      0.240836    0.00761865   -0.00941386    0.00798009       1.00027       Opttag_D
      59       82.2607       1.27836       1.00000  0.000000E+00       1.00000  0.000000E+00      0.941079       90.3154       1.00000       39.1502  0.000000E+00       9.42059  0.000000E+00       9.42059  0.000000E+00  0.000000E+00      -18.8412       83.8070       4.12606        1       Scatag_D
      59       1794.76       5462.95  0.000000E+00  0.000000E+00       2501.15  0.000000E+00       2556.79       8818.71  0.000000E+00       12665.2       49.1432       10858.3 -1.000000E+10 -1.000000E+10 -1.000000E+10 -1.000000E+10 -1.000000E+10  0.000000E+00  0.000000E+00  0.000000E+00  0.000000E+00  0.000000E+00  0.000000E+00  0.000000E+00  0.000000E+00  0.000000E+00  0.000000E+00  0.000000E+00  0.000000E+00       Funtag_D
      60  4.680634E+05  4.234060E+05       44657.4       10.5005     0.0548613      0.767759      0.188474      0.240820    0.00765294   -0.00938395    0.00797524      0.999412       Opttag_D
      60       82.2799       1.27867       1.00000  0.000000E+00       1.00000  0.000000E+00      0.941094       90.2943       1.00000       39.1591  0.000000E+00       9.41846  0.000000E+00       9.41846  0.000000E+00  0.000000E+00      -18.8369       83.8070       4.12606        1       Scatag_D
      60       1792.58       5453.55  0.000000E+00  0.000000E+00       2501.57  0.000000E+00       2556.88       8778.43  0.000000E+00       12667.5       49.1053       10857.8 -1.000000E+10 -1.000000E+10 -1.000000E+10 -1.000000E+10 -1.000000E+10  0.000000E+00  0.000000E+00  0.000000E+00  0.000000E+00  0.000000E+00  0.000000E+00  0.000000E+00  0.000000E+00  0.000000E+00  0.000000E+00  0.000000E+00  0.000000E+00       Funtag_D
      61  4.680296E+05  4.233943E+05       44635.3       9.18585     0.0552115      0.768923      0.188465      0.240799    0.00768349   -0.00934875    0.00797428      0.999299       Opttag_D
      61       82.2929       1.27879       1.00000  0.000000E+00       1.00000  0.000000E+00      0.941104       90.2803       1.00000       39.1653  0.000000E+00       9.41646  0.000000E+00       9.41646  0.000000E+00  0.000000E+00      -18.8329       83.8070       4.12606        1       Scatag_D
      61       1792.15       5452.32  0.000000E+00  0.000000E+00       2501.83  0.000000E+00       2556.36       8761.09  0.000000E+00       12668.3       49.0892       10854.1 -1.000000E+10 -1.000000E+10 -1.000000E+10 -1.000000E+10 -1.000000E+10  0.000000E+00  0.000000E+00  0.000000E+00  0.000000E+00  0.000000E+00  0.000000E+00  0.000000E+00  0.000000E+00  0.000000E+00  0.000000E+00  0.000000E+00  0.000000E+00       Funtag_D
      62  4.679859E+05  4.233687E+05       44617.2       11.2116     0.0561789      0.774332      0.188445      0.240749    0.00775073   -0.00924766    0.00797411      0.999293       Opttag_D
      62       82.3279       1.27927       1.00000  0.000000E+00       1.00000  0.000000E+00      0.941133       90.2419       1.00000       39.1821  0.000000E+00       9.41184  0.000000E+00       9.41184  0.000000E+00  0.000000E+00      -18.8237       83.8070       4.12606        1       Scatag_D
      62       1792.07       5452.26  0.000000E+00  0.000000E+00       2502.53  0.000000E+00       2555.95       8743.12  0.000000E+00       12671.5       49.0210       10850.8 -1.000000E+10 -1.000000E+10 -1.000000E+10 -1.000000E+10 -1.000000E+10  0.000000E+00  0.000000E+00  0.000000E+00  0.000000E+00  0.000000E+00  0.000000E+00  0.000000E+00  0.000000E+00  0.000000E+00  0.000000E+00  0.000000E+00  0.000000E+00       Funtag_D
      63  4.679530E+05  4.233230E+05       44630.1       25.8437     0.0583024      0.807029      0.188405      0.240643    0.00787033   -0.00902106    0.00799195       1.00033       Opttag_D
      63       82.4035       1.28058       1.00000  0.000000E+00       1.00000  0.000000E+00      0.941189       90.1633       1.00000       39.2178  0.000000E+00       9.40185  0.000000E+00       9.40185  0.000000E+00  0.000000E+00      -18.8037       83.8070       4.12606        1       Scatag_D
      63       1800.10       5463.58  0.000000E+00  0.000000E+00       2503.77  0.000000E+00       2555.92       8729.17  0.000000E+00       12678.8       48.8368       10849.8 -1.000000E+10 -1.000000E+10 -1.000000E+10 -1.000000E+10 -1.000000E+10  0.000000E+00  0.000000E+00  0.000000E+00  0.000000E+00  0.000000E+00  0.000000E+00  0.000000E+00  0.000000E+00  0.000000E+00  0.000000E+00  0.000000E+00  0.000000E+00       Funtag_D
      64  4.678893E+05  4.233057E+05       44583.6       11.7417     0.0591487      0.831655      0.188390      0.240611    0.00791557   -0.00893289    0.00798059      0.999363       Opttag_D
      64       82.4328       1.28105       1.00000  0.000000E+00       1.00000  0.000000E+00      0.941213       90.1291       1.00000       39.2331  0.000000E+00       9.39843  0.000000E+00       9.39843  0.000000E+00  0.000000E+00      -18.7969       83.8070       4.12606        1       Scatag_D
      64       1794.99       5453.02  0.000000E+00  0.000000E+00       2504.22  0.000000E+00       2556.17       8693.49  0.000000E+00       12682.1       48.7674       10850.8 -1.000000E+10 -1.000000E+10 -1.000000E+10 -1.000000E+10 -1.000000E+10  0.000000E+00  0.000000E+00  0.000000E+00  0.000000E+00  0.000000E+00  0.000000E+00  0.000000E+00  0.000000E+00  0.000000E+00  0.000000E+00  0.000000E+00  0.000000E+00       Funtag_D
      65  4.678504E+05  4.232885E+05       44561.9       8.83575     0.0600034      0.857145      0.188372      0.240577    0.00796072   -0.00883652    0.00797796      0.999334       Opttag_D
      65       82.4625       1.28152       1.00000  0.000000E+00       1.00000  0.000000E+00      0.941237       90.0950       1.00000       39.2474  0.000000E+00       9.39497  0.000000E+00       9.39497  0.000000E+00  0.000000E+00      -18.7899       83.8070       4.12606        1       Scatag_D
      65       1793.80       5452.71  0.000000E+00  0.000000E+00       2504.58  0.000000E+00       2556.37       8670.60  0.000000E+00       12685.1       48.6807       10850.1 -1.000000E+10 -1.000000E+10 -1.000000E+10 -1.000000E+10 -1.000000E+10  0.000000E+00  0.000000E+00  0.000000E+00  0.000000E+00  0.000000E+00  0.000000E+00  0.000000E+00  0.000000E+00  0.000000E+00  0.000000E+00  0.000000E+00  0.000000E+00       Funtag_D
      66  4.678099E+05  4.232633E+05       44546.6       10.9190     0.0613436      0.897221      0.188348      0.240525    0.00802672   -0.00869482    0.00797840      0.999407       Opttag_D
      66       82.5086       1.28227       1.00000  0.000000E+00       1.00000  0.000000E+00      0.941270       90.0424       1.00000       39.2705  0.000000E+00       9.38933  0.000000E+00       9.38933  0.000000E+00  0.000000E+00      -18.7787       83.8070       4.12606        1       Scatag_D
      66       1794.00       5453.50  0.000000E+00  0.000000E+00       2505.06  0.000000E+00       2556.63       8649.82  0.000000E+00       12689.9       48.5630       10849.1 -1.000000E+10 -1.000000E+10 -1.000000E+10 -1.000000E+10 -1.000000E+10  0.000000E+00  0.000000E+00  0.000000E+00  0.000000E+00  0.000000E+00  0.000000E+00  0.000000E+00  0.000000E+00  0.000000E+00  0.000000E+00  0.000000E+00  0.000000E+00       Funtag_D
      67  4.677561E+05  4.232258E+05       44530.3       17.8416     0.0636689      0.967051      0.188311      0.240440    0.00812480   -0.00842257    0.00798027      0.999863       Opttag_D
      67       82.5869       1.28355       1.00000  0.000000E+00       1.00000  0.000000E+00      0.941323       89.9473       1.00000       39.3128  0.000000E+00       9.37928  0.000000E+00       9.37928  0.000000E+00  0.000000E+00      -18.7586       83.8070       4.12606        1       Scatag_D
      67       1794.84       5458.48  0.000000E+00  0.000000E+00       2505.71  0.000000E+00       2556.65       8620.95  0.000000E+00       12698.6       48.4536       10846.6 -1.000000E+10 -1.000000E+10 -1.000000E+10 -1.000000E+10 -1.000000E+10  0.000000E+00  0.000000E+00  0.000000E+00  0.000000E+00  0.000000E+00  0.000000E+00  0.000000E+00  0.000000E+00  0.000000E+00  0.000000E+00  0.000000E+00  0.000000E+00       Funtag_D
      68  4.677039E+05  4.231941E+05       44509.8       11.8865     0.0661592       1.03997      0.188275      0.240370    0.00820779   -0.00820474    0.00798597      0.999398       Opttag_D
      68       82.6684       1.28499       1.00000  0.000000E+00       1.00000  0.000000E+00      0.941375       89.8472       1.00000       39.3626  0.000000E+00       9.36866  0.000000E+00       9.36866  0.000000E+00  0.000000E+00      -18.7373       83.8070       4.12606        1       Scatag_D
      68       1797.41       5453.40  0.000000E+00  0.000000E+00       2506.25  0.000000E+00       2556.95       8589.77  0.000000E+00       12709.4       48.3722       10848.2 -1.000000E+10 -1.000000E+10 -1.000000E+10 -1.000000E+10 -1.000000E+10  0.000000E+00  0.000000E+00  0.000000E+00  0.000000E+00  0.000000E+00  0.000000E+00  0.000000E+00  0.000000E+00  0.000000E+00  0.000000E+00  0.000000E+00  0.000000E+00       Funtag_D
      69  4.676700E+05  4.231860E+05       44484.0       8.48822     0.0664359       1.04968      0.188270      0.240358    0.00822912   -0.00818045    0.00798645      0.999198       Opttag_D
      69       82.6767       1.28489       1.00000  0.000000E+00       1.00000  0.000000E+00      0.941375       89.8314       1.00000       39.3678  0.000000E+00       9.36724  0.000000E+00       9.36724  0.000000E+00  0.000000E+00      -18.7345       83.8070       4.12606        1       Scatag_D
      69       1797.63       5451.22  0.000000E+00  0.000000E+00       2506.22  0.000000E+00       2556.00       8570.61  0.000000E+00       12709.6       48.4333       10844.3 -1.000000E+10 -1.000000E+10 -1.000000E+10 -1.000000E+10 -1.000000E+10  0.000000E+00  0.000000E+00  0.000000E+00  0.000000E+00  0.000000E+00  0.000000E+00  0.000000E+00  0.000000E+00  0.000000E+00  0.000000E+00  0.000000E+00  0.000000E+00       Funtag_D
      70  4.676232E+05  4.231662E+05       44457.0       10.6837     0.0671632       1.07448      0.188257      0.240318    0.00828090   -0.00810185    0.00798903      0.999137       Opttag_D
      70       82.6977       1.28458       1.00000  0.000000E+00       1.00000  0.000000E+00      0.941375       89.7884       1.00000       39.3813  0.000000E+00       9.36380  0.000000E+00       9.36380  0.000000E+00  0.000000E+00      -18.7276       83.8070       4.12606        1       Scatag_D
      70       1798.79       5450.56  0.000000E+00  0.000000E+00       2506.15  0.000000E+00       2553.80       8551.52  0.000000E+00       12710.1       48.6044       10837.5 -1.000000E+10 -1.000000E+10 -1.000000E+10 -1.000000E+10 -1.000000E+10  0.000000E+00  0.000000E+00  0.000000E+00  0.000000E+00  0.000000E+00  0.000000E+00  0.000000E+00  0.000000E+00  0.000000E+00  0.000000E+00  0.000000E+00  0.000000E+00       Funtag_D
      71  4.675936E+05  4.231432E+05       44450.5       15.5998     0.0686984       1.12125      0.188236      0.240260    0.00834122   -0.00798017    0.00799570      0.999233       Opttag_D
      71       82.7422       1.28477       1.00000  0.000000E+00       1.00000  0.000000E+00      0.941393       89.7133       1.00000       39.4107  0.000000E+00       9.35813  0.000000E+00       9.35813  0.000000E+00  0.000000E+00      -18.7163       83.8070       4.12606        1       Scatag_D
      71       1801.79       5451.60  0.000000E+00  0.000000E+00       2506.26  0.000000E+00       2552.05       8540.60  0.000000E+00       12714.6       48.7533       10834.8 -1.000000E+10 -1.000000E+10 -1.000000E+10 -1.000000E+10 -1.000000E+10  0.000000E+00  0.000000E+00  0.000000E+00  0.000000E+00  0.000000E+00  0.000000E+00  0.000000E+00  0.000000E+00  0.000000E+00  0.000000E+00  0.000000E+00  0.000000E+00       Funtag_D
      72  4.675568E+05  4.231327E+05       44424.2       9.43131     0.0694246       1.14274      0.188225      0.240231    0.00836879   -0.00792372    0.00799717      0.999321       Opttag_D
      72       82.7621       1.28480       1.00000  0.000000E+00       1.00000  0.000000E+00      0.941403       89.6763       1.00000       39.4239  0.000000E+00       9.35572  0.000000E+00       9.35572  0.000000E+00  0.000000E+00      -18.7114       83.8070       4.12606        1       Scatag_D
      72       1802.46       5452.56  0.000000E+00  0.000000E+00       2506.32  0.000000E+00       2551.35       8512.57  0.000000E+00       12716.7       48.8155       10833.3 -1.000000E+10 -1.000000E+10 -1.000000E+10 -1.000000E+10 -1.000000E+10  0.000000E+00  0.000000E+00  0.000000E+00  0.000000E+00  0.000000E+00  0.000000E+00  0.000000E+00  0.000000E+00  0.000000E+00  0.000000E+00  0.000000E+00  0.000000E+00       Funtag_D
      73  4.675137E+05  4.231155E+05       44398.2       8.64140     0.0708859       1.18441      0.188206      0.240186    0.00841365   -0.00782566    0.00800049      0.999592       Opttag_D
      73       82.8012       1.28499       1.00000  0.000000E+00       1.00000  0.000000E+00      0.941426       89.6055       1.00000       39.4501  0.000000E+00       9.35143  0.000000E+00       9.35143  0.000000E+00  0.000000E+00      -18.7029       83.8070       4.12606        1       Scatag_D
      73       1803.95       5455.52  0.000000E+00  0.000000E+00       2506.58  0.000000E+00       2550.55       8480.55  0.000000E+00       12721.5       48.8852       10830.7 -1.000000E+10 -1.000000E+10 -1.000000E+10 -1.000000E+10 -1.000000E+10  0.000000E+00  0.000000E+00  0.000000E+00  0.000000E+00  0.000000E+00  0.000000E+00  0.000000E+00  0.000000E+00  0.000000E+00  0.000000E+00  0.000000E+00  0.000000E+00       Funtag_D
      74  4.674822E+05  4.231011E+05       44381.1       9.74617     0.0722106       1.22107      0.188190      0.240154    0.00845150   -0.00776628    0.00800445      0.999772       Opttag_D
      74       82.8354       1.28508       1.00000  0.000000E+00       1.00000  0.000000E+00      0.941451       89.5395       1.00000       39.4745  0.000000E+00       9.34814  0.000000E+00       9.34814  0.000000E+00  0.000000E+00      -18.6963       83.8070       4.12606        1       Scatag_D
      74       1805.74       5457.49  0.000000E+00  0.000000E+00       2506.89  0.000000E+00       2550.32       8457.33  0.000000E+00       12726.2       48.9266       10828.2 -1.000000E+10 -1.000000E+10 -1.000000E+10 -1.000000E+10 -1.000000E+10  0.000000E+00  0.000000E+00  0.000000E+00  0.000000E+00  0.000000E+00  0.000000E+00  0.000000E+00  0.000000E+00  0.000000E+00  0.000000E+00  0.000000E+00  0.000000E+00       Funtag_D
      75  4.674516E+05  4.230721E+05       44379.6       18.6147     0.0755978       1.31139      0.188160      0.240084    0.00852749   -0.00762335    0.00801527       1.00052       Opttag_D
      75       82.9194       1.28528       1.00000  0.000000E+00       1.00000  0.000000E+00      0.941518       89.3654       1.00000       39.5408  0.000000E+00       9.34030  0.000000E+00       9.34030  0.000000E+00  0.000000E+00      -18.6806       83.8070       4.12606        1       Scatag_D
      75       1810.62       5465.62  0.000000E+00  0.000000E+00       2507.91  0.000000E+00       2551.10       8429.74  0.000000E+00       12739.9       49.0291       10825.6 -1.000000E+10 -1.000000E+10 -1.000000E+10 -1.000000E+10 -1.000000E+10  0.000000E+00  0.000000E+00  0.000000E+00  0.000000E+00  0.000000E+00  0.000000E+00  0.000000E+00  0.000000E+00  0.000000E+00  0.000000E+00  0.000000E+00  0.000000E+00       Funtag_D
      76  4.674062E+05  4.230662E+05       44340.0       8.11896     0.0758161       1.31564      0.188158      0.240091    0.00854286   -0.00764809    0.00801315       1.00032       Opttag_D
      76       82.9230       1.28497       1.00000  0.000000E+00       1.00000  0.000000E+00      0.941528       89.3472       1.00000       39.5448  0.000000E+00       9.34089  0.000000E+00       9.34089  0.000000E+00  0.000000E+00      -18.6818       83.8070       4.12606        1       Scatag_D
      76       1809.67       5463.48  0.000000E+00  0.000000E+00       2508.12  0.000000E+00       2551.44       8394.95  0.000000E+00       12740.3       49.0091       10823.0 -1.000000E+10 -1.000000E+10 -1.000000E+10 -1.000000E+10 -1.000000E+10  0.000000E+00  0.000000E+00  0.000000E+00  0.000000E+00  0.000000E+00  0.000000E+00  0.000000E+00  0.000000E+00  0.000000E+00  0.000000E+00  0.000000E+00  0.000000E+00       Funtag_D
      77  4.673838E+05  4.230596E+05       44324.2       7.63958     0.0758254       1.31390      0.188157      0.240098    0.00856005   -0.00767733    0.00801043       1.00015       Opttag_D
      77       82.9210       1.28453       1.00000  0.000000E+00       1.00000  0.000000E+00      0.941535       89.3369       1.00000       39.5445  0.000000E+00       9.34202  0.000000E+00       9.34202  0.000000E+00  0.000000E+00      -18.6840       83.8070       4.12606        1       Scatag_D
      77       1808.44       5461.60  0.000000E+00  0.000000E+00       2508.28  0.000000E+00       2551.66       8385.50  0.000000E+00       12739.5       48.9909       10820.2 -1.000000E+10 -1.000000E+10 -1.000000E+10 -1.000000E+10 -1.000000E+10  0.000000E+00  0.000000E+00  0.000000E+00  0.000000E+00  0.000000E+00  0.000000E+00  0.000000E+00  0.000000E+00  0.000000E+00  0.000000E+00  0.000000E+00  0.000000E+00       Funtag_D
      78  4.673497E+05  4.230437E+05       44306.1       9.58339     0.0767195       1.33248      0.188150      0.240100    0.00860193   -0.00771343    0.00801052       1.00014       Opttag_D
      78       82.9386       1.28382       1.00000  0.000000E+00       1.00000  0.000000E+00      0.941564       89.2708       1.00000       39.5627  0.000000E+00       9.34233  0.000000E+00       9.34233  0.000000E+00  0.000000E+00      -18.6847       83.8070       4.12606        1       Scatag_D
      78       1808.48       5461.50  0.000000E+00  0.000000E+00       2508.88  0.000000E+00       2552.21       8367.20  0.000000E+00       12741.8       49.0232       10817.0 -1.000000E+10 -1.000000E+10 -1.000000E+10 -1.000000E+10 -1.000000E+10  0.000000E+00  0.000000E+00  0.000000E+00  0.000000E+00  0.000000E+00  0.000000E+00  0.000000E+00  0.000000E+00  0.000000E+00  0.000000E+00  0.000000E+00  0.000000E+00       Funtag_D
      79  4.673463E+05  4.230247E+05       44321.6       26.6637     0.0793023       1.38548      0.188137      0.240104    0.00865150   -0.00777089    0.00802120       1.00054       Opttag_D
      79       82.9919       1.28291       1.00000  0.000000E+00       1.00000  0.000000E+00      0.941639       89.0958       1.00000       39.6162  0.000000E+00       9.34178  0.000000E+00       9.34178  0.000000E+00  0.000000E+00      -18.6836       83.8070       4.12606        1       Scatag_D
      79       1813.30       5465.83  0.000000E+00  0.000000E+00       2510.34  0.000000E+00       2553.11       8362.48  0.000000E+00       12750.2       49.2136       10817.1 -1.000000E+10 -1.000000E+10 -1.000000E+10 -1.000000E+10 -1.000000E+10  0.000000E+00  0.000000E+00  0.000000E+00  0.000000E+00  0.000000E+00  0.000000E+00  0.000000E+00  0.000000E+00  0.000000E+00  0.000000E+00  0.000000E+00  0.000000E+00       Funtag_D
      80  4.672870E+05  4.230216E+05       44265.4       9.46071     0.0794987       1.38980      0.188137      0.240102    0.00865967   -0.00778585    0.00801970       1.00049       Opttag_D
      80       82.9967       1.28284       1.00000  0.000000E+00       1.00000  0.000000E+00      0.941643       89.0834       1.00000       39.6209  0.000000E+00       9.34147  0.000000E+00       9.34147  0.000000E+00  0.000000E+00      -18.6829       83.8070       4.12606        1       Scatag_D
      80       1812.62       5465.34  0.000000E+00  0.000000E+00       2510.40  0.000000E+00       2552.97       8307.04  0.000000E+00       12750.8       49.2384       10817.0 -1.000000E+10 -1.000000E+10 -1.000000E+10 -1.000000E+10 -1.000000E+10  0.000000E+00  0.000000E+00  0.000000E+00  0.000000E+00  0.000000E+00  0.000000E+00  0.000000E+00  0.000000E+00  0.000000E+00  0.000000E+00  0.000000E+00  0.000000E+00       Funtag_D
      81  4.672686E+05  4.230200E+05       44248.5       6.30302     0.0801085       1.40232      0.188133      0.240101    0.00866380   -0.00779125    0.00802084       1.00058       Opttag_D
      81       83.0105       1.28282       1.00000  0.000000E+00       1.00000  0.000000E+00      0.941657       89.0458       1.00000       39.6340  0.000000E+00       9.34076  0.000000E+00       9.34076  0.000000E+00  0.000000E+00      -18.6815       83.8070       4.12606        1       Scatag_D
      81       1813.14       5466.27  0.000000E+00  0.000000E+00       2510.63  0.000000E+00       2552.67       8286.76  0.000000E+00       12752.9       49.3118       10816.8 -1.000000E+10 -1.000000E+10 -1.000000E+10 -1.000000E+10 -1.000000E+10  0.000000E+00  0.000000E+00  0.000000E+00  0.000000E+00  0.000000E+00  0.000000E+00  0.000000E+00  0.000000E+00  0.000000E+00  0.000000E+00  0.000000E+00  0.000000E+00       Funtag_D
      82  4.672455E+05  4.230184E+05       44227.2       7.04377     0.0814702       1.42832      0.188129      0.240103    0.00866812   -0.00781081    0.00802480       1.00076       Opttag_D
      82       83.0401       1.28272       1.00000  0.000000E+00       1.00000  0.000000E+00      0.941692       88.9586       1.00000       39.6635  0.000000E+00       9.33970  0.000000E+00       9.33970  0.000000E+00  0.000000E+00      -18.6794       83.8070       4.12606        1       Scatag_D
      82       1814.93       5468.33  0.000000E+00  0.000000E+00       2511.18  0.000000E+00       2552.34       8255.85  0.000000E+00       12757.8       49.4740       10817.3 -1.000000E+10 -1.000000E+10 -1.000000E+10 -1.000000E+10 -1.000000E+10  0.000000E+00  0.000000E+00  0.000000E+00  0.000000E+00  0.000000E+00  0.000000E+00  0.000000E+00  0.000000E+00  0.000000E+00  0.000000E+00  0.000000E+00  0.000000E+00       Funtag_D
      83  4.672147E+05  4.230167E+05       44198.0       8.26838     0.0831195       1.45463      0.188126      0.240107    0.00867254   -0.00781048    0.00802627       1.00090       Opttag_D
      83       83.0754       1.28254       1.00000  0.000000E+00       1.00000  0.000000E+00      0.941734       88.8487       1.00000       39.6993  0.000000E+00       9.33854  0.000000E+00       9.33854  0.000000E+00  0.000000E+00      -18.6771       83.8070       4.12606        1       Scatag_D
      83       1815.59       5469.77  0.000000E+00  0.000000E+00       2511.80  0.000000E+00       2552.24       8218.00  0.000000E+00       12763.3       49.6621       10817.7 -1.000000E+10 -1.000000E+10 -1.000000E+10 -1.000000E+10 -1.000000E+10  0.000000E+00  0.000000E+00  0.000000E+00  0.000000E+00  0.000000E+00  0.000000E+00  0.000000E+00  0.000000E+00  0.000000E+00  0.000000E+00  0.000000E+00  0.000000E+00       Funtag_D

 An atom has moved more than     1.50 A from the initial/last
 position - if this was an accepted trial point, probably need to update the
 non-bonded contact list.

      84  4.672094E+05  4.230287E+05       44180.7       19.3076     0.0867932       1.50678      0.188133      0.240156    0.00864103   -0.00787379    0.00803317       1.00135       Opttag_D
      84       83.1532       1.28253       1.00000  0.000000E+00       1.00000  0.000000E+00      0.941826       88.6072       1.00000       39.7821  0.000000E+00       9.33666  0.000000E+00       9.33666  0.000000E+00  0.000000E+00      -18.6733       83.8070       4.12606        1       Scatag_D
      84       1818.72       5474.71  0.000000E+00  0.000000E+00       2513.29  0.000000E+00       2553.28       8170.53  0.000000E+00       12776.7       50.0285       10823.5 -1.000000E+10 -1.000000E+10 -1.000000E+10 -1.000000E+10 -1.000000E+10  0.000000E+00  0.000000E+00  0.000000E+00  0.000000E+00  0.000000E+00  0.000000E+00  0.000000E+00  0.000000E+00  0.000000E+00  0.000000E+00  0.000000E+00  0.000000E+00       Funtag_D

 An atom has moved more than     1.50 A from the initial/last
 position - need to update the non-bonded contact list.


 ############################################################################################################
 ###################################################### setting up contact terms
 ############################################################################################################

      85  4.671674E+05  4.230187E+05       44148.6       6.08857     0.0860712       1.49106      0.188128      0.240144    0.00866726   -0.00782622    0.00802814       1.00109       Opttag_D
      85       83.1376       1.28226       1.00000  0.000000E+00       1.00000  0.000000E+00      0.941809       88.6491       1.00000       39.7643  0.000000E+00       9.33713  0.000000E+00       9.33713  0.000000E+00  0.000000E+00      -18.6743       83.8070       4.12606        1       Scatag_D
      85       1816.44       5471.89  0.000000E+00  0.000000E+00       2512.90  0.000000E+00       2553.17       8151.11  0.000000E+00       12772.4       49.9247       10820.8 -1.000000E+10 -1.000000E+10 -1.000000E+10 -1.000000E+10 -1.000000E+10  0.000000E+00  0.000000E+00  0.000000E+00  0.000000E+00  0.000000E+00  0.000000E+00  0.000000E+00  0.000000E+00  0.000000E+00  0.000000E+00  0.000000E+00  0.000000E+00       Funtag_D
      86  4.671551E+05  4.230121E+05       44143.0       5.74865     0.0858264       1.48186      0.188126      0.240143    0.00868445   -0.00780879    0.00802510       1.00095       Opttag_D
      86       83.1324       1.28206       1.00000  0.000000E+00       1.00000  0.000000E+00      0.941803       88.6610       1.00000       39.7575  0.000000E+00       9.33744  0.000000E+00       9.33744  0.000000E+00  0.000000E+00      -18.6749       83.8070       4.12606        1       Scatag_D
      86       1815.07       5470.36  0.000000E+00  0.000000E+00       2512.73  0.000000E+00       2553.29       8152.37  0.000000E+00       12769.9       49.8626       10819.5 -1.000000E+10 -1.000000E+10 -1.000000E+10 -1.000000E+10 -1.000000E+10  0.000000E+00  0.000000E+00  0.000000E+00  0.000000E+00  0.000000E+00  0.000000E+00  0.000000E+00  0.000000E+00  0.000000E+00  0.000000E+00  0.000000E+00  0.000000E+00       Funtag_D
      87  4.671276E+05  4.229973E+05       44130.2       8.06174     0.0862419       1.47021      0.188118      0.240157    0.00872316   -0.00779777    0.00802084       1.00080       Opttag_D
      87       83.1412       1.28154       1.00000  0.000000E+00       1.00000  0.000000E+00      0.941813       88.6241       1.00000       39.7634  0.000000E+00       9.33758  0.000000E+00       9.33758  0.000000E+00  0.000000E+00      -18.6752       83.8070       4.12606        1       Scatag_D
      87       1813.14       5468.70  0.000000E+00  0.000000E+00       2512.76  0.000000E+00       2554.07       8146.02  0.000000E+00       12767.0       49.7975       10818.8 -1.000000E+10 -1.000000E+10 -1.000000E+10 -1.000000E+10 -1.000000E+10  0.000000E+00  0.000000E+00  0.000000E+00  0.000000E+00  0.000000E+00  0.000000E+00  0.000000E+00  0.000000E+00  0.000000E+00  0.000000E+00  0.000000E+00  0.000000E+00       Funtag_D
      88  4.671122E+05  4.229823E+05       44129.9       18.3367     0.0876696       1.45819      0.188109      0.240190    0.00876255   -0.00780182    0.00802035       1.00084       Opttag_D
      88       83.1722       1.28104       1.00000  0.000000E+00       1.00000  0.000000E+00      0.941844       88.5222       1.00000       39.7890  0.000000E+00       9.33682  0.000000E+00       9.33682  0.000000E+00  0.000000E+00      -18.6736       83.8070       4.12606        1       Scatag_D
      88       1812.92       5469.17  0.000000E+00  0.000000E+00       2513.21  0.000000E+00       2555.62       8142.74  0.000000E+00       12765.2       49.7973       10821.2 -1.000000E+10 -1.000000E+10 -1.000000E+10 -1.000000E+10 -1.000000E+10  0.000000E+00  0.000000E+00  0.000000E+00  0.000000E+00  0.000000E+00  0.000000E+00  0.000000E+00  0.000000E+00  0.000000E+00  0.000000E+00  0.000000E+00  0.000000E+00       Funtag_D
      89  4.670803E+05  4.229790E+05       44101.3       8.68936     0.0882044       1.45771      0.188108      0.240206    0.00877129   -0.00783263    0.00802090       1.00088       Opttag_D
      89       83.1839       1.28094       1.00000  0.000000E+00       1.00000  0.000000E+00      0.941857       88.4857       1.00000       39.7996  0.000000E+00       9.33646  0.000000E+00       9.33646  0.000000E+00  0.000000E+00      -18.6729       83.8070       4.12606        1       Scatag_D
      89       1813.17       5469.59  0.000000E+00  0.000000E+00       2513.46  0.000000E+00       2556.00       8111.40  0.000000E+00       12765.7       49.8301       10822.2 -1.000000E+10 -1.000000E+10 -1.000000E+10 -1.000000E+10 -1.000000E+10  0.000000E+00  0.000000E+00  0.000000E+00  0.000000E+00  0.000000E+00  0.000000E+00  0.000000E+00  0.000000E+00  0.000000E+00  0.000000E+00  0.000000E+00  0.000000E+00       Funtag_D
      90  4.670621E+05  4.229773E+05       44084.8       6.09834     0.0890248       1.45887      0.188105      0.240224    0.00877571   -0.00786940    0.00802211       1.00098       Opttag_D
      90       83.2021       1.28094       1.00000  0.000000E+00       1.00000  0.000000E+00      0.941877       88.4332       1.00000       39.8159  0.000000E+00       9.33564  0.000000E+00       9.33564  0.000000E+00  0.000000E+00      -18.6713       83.8070       4.12606        1       Scatag_D
      90       1813.71       5470.64  0.000000E+00  0.000000E+00       2513.87  0.000000E+00       2556.26       8090.75  0.000000E+00       12767.0       49.9067       10822.6 -1.000000E+10 -1.000000E+10 -1.000000E+10 -1.000000E+10 -1.000000E+10  0.000000E+00  0.000000E+00  0.000000E+00  0.000000E+00  0.000000E+00  0.000000E+00  0.000000E+00  0.000000E+00  0.000000E+00  0.000000E+00  0.000000E+00  0.000000E+00       Funtag_D
      91  4.670450E+05  4.229742E+05       44070.8       6.49095     0.0900247       1.45579      0.188102      0.240244    0.00878378   -0.00790145    0.00802311       1.00110       Opttag_D
      91       83.2246       1.28097       1.00000  0.000000E+00       1.00000  0.000000E+00      0.941900       88.3696       1.00000       39.8343  0.000000E+00       9.33442  0.000000E+00       9.33442  0.000000E+00  0.000000E+00      -18.6688       83.8070       4.12606        1       Scatag_D
      91       1814.17       5472.01  0.000000E+00  0.000000E+00       2514.39  0.000000E+00       2556.59       8072.79  0.000000E+00       12767.9       49.9957       10822.9 -1.000000E+10 -1.000000E+10 -1.000000E+10 -1.000000E+10 -1.000000E+10  0.000000E+00  0.000000E+00  0.000000E+00  0.000000E+00  0.000000E+00  0.000000E+00  0.000000E+00  0.000000E+00  0.000000E+00  0.000000E+00  0.000000E+00  0.000000E+00       Funtag_D
      92  4.670177E+05  4.229661E+05       44051.6       9.09411     0.0915824       1.44422      0.188091      0.240273    0.00880510   -0.00793654    0.00802197       1.00128       Opttag_D
      92       83.2610       1.28111       1.00000  0.000000E+00       1.00000  0.000000E+00      0.941933       88.2752       1.00000       39.8595  0.000000E+00       9.33217  0.000000E+00       9.33217  0.000000E+00  0.000000E+00      -18.6643       83.8070       4.12606        1       Scatag_D
      92       1813.65       5473.97  0.000000E+00  0.000000E+00       2515.29  0.000000E+00       2556.71       8052.00  0.000000E+00       12768.4       50.0806       10821.5 -1.000000E+10 -1.000000E+10 -1.000000E+10 -1.000000E+10 -1.000000E+10  0.000000E+00  0.000000E+00  0.000000E+00  0.000000E+00  0.000000E+00  0.000000E+00  0.000000E+00  0.000000E+00  0.000000E+00  0.000000E+00  0.000000E+00  0.000000E+00       Funtag_D
      93  4.670011E+05  4.229603E+05       44040.8       12.0133     0.0939520       1.42289      0.188077      0.240313    0.00882019   -0.00798208    0.00802388       1.00165       Opttag_D
      93       83.3161       1.28166       1.00000  0.000000E+00       1.00000  0.000000E+00      0.941979       88.1394       1.00000       39.8966  0.000000E+00       9.32844  0.000000E+00       9.32844  0.000000E+00  0.000000E+00      -18.6569       83.8070       4.12606        1       Scatag_D
      93       1814.52       5477.97  0.000000E+00  0.000000E+00       2516.76  0.000000E+00       2557.09       8032.64  0.000000E+00       12769.8       50.1713       10821.9 -1.000000E+10 -1.000000E+10 -1.000000E+10 -1.000000E+10 -1.000000E+10  0.000000E+00  0.000000E+00  0.000000E+00  0.000000E+00  0.000000E+00  0.000000E+00  0.000000E+00  0.000000E+00  0.000000E+00  0.000000E+00  0.000000E+00  0.000000E+00       Funtag_D
      94  4.669792E+05  4.229519E+05       44027.3       6.11703     0.0935683       1.42003      0.188071      0.240307    0.00884209   -0.00797421    0.00802136       1.00151       Opttag_D
      94       83.3088       1.28155       1.00000  0.000000E+00       1.00000  0.000000E+00      0.941967       88.1666       1.00000       39.8851  0.000000E+00       9.32876  0.000000E+00       9.32876  0.000000E+00  0.000000E+00      -18.6575       83.8070       4.12606        1       Scatag_D
      94       1813.38       5476.53  0.000000E+00  0.000000E+00       2516.59  0.000000E+00       2556.39       8026.49  0.000000E+00       12767.9       50.0904       10819.9 -1.000000E+10 -1.000000E+10 -1.000000E+10 -1.000000E+10 -1.000000E+10  0.000000E+00  0.000000E+00  0.000000E+00  0.000000E+00  0.000000E+00  0.000000E+00  0.000000E+00  0.000000E+00  0.000000E+00  0.000000E+00  0.000000E+00  0.000000E+00       Funtag_D
      95  4.669630E+05  4.229386E+05       44024.5       6.44362     0.0933657       1.41267      0.188059      0.240304    0.00887715   -0.00797174    0.00801904       1.00141       Opttag_D
      95       83.3066       1.28149       1.00000  0.000000E+00       1.00000  0.000000E+00      0.941953       88.1885       1.00000       39.8734  0.000000E+00       9.32865  0.000000E+00       9.32865  0.000000E+00  0.000000E+00      -18.6573       83.8070       4.12606        1       Scatag_D
      95       1812.33       5475.42  0.000000E+00  0.000000E+00       2516.58  0.000000E+00       2555.43       8031.29  0.000000E+00       12765.5       49.9790       10817.9 -1.000000E+10 -1.000000E+10 -1.000000E+10 -1.000000E+10 -1.000000E+10  0.000000E+00  0.000000E+00  0.000000E+00  0.000000E+00  0.000000E+00  0.000000E+00  0.000000E+00  0.000000E+00  0.000000E+00  0.000000E+00  0.000000E+00  0.000000E+00       Funtag_D
      96  4.669416E+05  4.229161E+05       44025.4       7.91398     0.0937622       1.39620      0.188036      0.240318    0.00893584   -0.00801807    0.00801708       1.00144       Opttag_D
      96       83.3197       1.28161       1.00000  0.000000E+00       1.00000  0.000000E+00      0.941942       88.1860       1.00000       39.8661  0.000000E+00       9.32743  0.000000E+00       9.32743  0.000000E+00  0.000000E+00      -18.6549       83.8070       4.12606        1       Scatag_D
      96       1811.44       5475.66  0.000000E+00  0.000000E+00       2517.01  0.000000E+00       2554.16       8037.39  0.000000E+00       12762.9       49.8396       10817.1 -1.000000E+10 -1.000000E+10 -1.000000E+10 -1.000000E+10 -1.000000E+10  0.000000E+00  0.000000E+00  0.000000E+00  0.000000E+00  0.000000E+00  0.000000E+00  0.000000E+00  0.000000E+00  0.000000E+00  0.000000E+00  0.000000E+00  0.000000E+00       Funtag_D
      97  4.669278E+05  4.228762E+05       44051.6       16.0568     0.0950579       1.36230      0.187990      0.240344    0.00904036   -0.00809667    0.00801619       1.00167       Opttag_D
      97       83.3570       1.28224       1.00000  0.000000E+00       1.00000  0.000000E+00      0.941919       88.1647       1.00000       39.8593  0.000000E+00       9.32380  0.000000E+00       9.32380  0.000000E+00  0.000000E+00      -18.6476       83.8070       4.12606        1       Scatag_D
      97       1811.04       5478.24  0.000000E+00  0.000000E+00       2518.15  0.000000E+00       2552.07       8061.83  0.000000E+00       12760.3       49.6394       10820.3 -1.000000E+10 -1.000000E+10 -1.000000E+10 -1.000000E+10 -1.000000E+10  0.000000E+00  0.000000E+00  0.000000E+00  0.000000E+00  0.000000E+00  0.000000E+00  0.000000E+00  0.000000E+00  0.000000E+00  0.000000E+00  0.000000E+00  0.000000E+00       Funtag_D
      98  4.669011E+05  4.228704E+05       44030.7       7.09263     0.0954774       1.35749      0.187984      0.240362    0.00905563   -0.00815841    0.00801560       1.00168       Opttag_D
      98       83.3676       1.28239       1.00000  0.000000E+00       1.00000  0.000000E+00      0.941919       88.1499       1.00000       39.8625  0.000000E+00       9.32302  0.000000E+00       9.32302  0.000000E+00  0.000000E+00      -18.6460       83.8070       4.12606        1       Scatag_D
      98       1810.77       5478.30  0.000000E+00  0.000000E+00       2518.45  0.000000E+00       2551.83       8039.97  0.000000E+00       12760.9       49.6512       10820.9 -1.000000E+10 -1.000000E+10 -1.000000E+10 -1.000000E+10 -1.000000E+10  0.000000E+00  0.000000E+00  0.000000E+00  0.000000E+00  0.000000E+00  0.000000E+00  0.000000E+00  0.000000E+00  0.000000E+00  0.000000E+00  0.000000E+00  0.000000E+00       Funtag_D
      99  4.668884E+05  4.228648E+05       44023.6       5.62031     0.0959813       1.35328      0.187975      0.240377    0.00907043   -0.00820666    0.00801487       1.00168       Opttag_D
      99       83.3802       1.28259       1.00000  0.000000E+00       1.00000  0.000000E+00      0.941918       88.1331       1.00000       39.8666  0.000000E+00       9.32197  0.000000E+00       9.32197  0.000000E+00  0.000000E+00      -18.6439       83.8070       4.12606        1       Scatag_D
      99       1810.44       5478.33  0.000000E+00  0.000000E+00       2518.78  0.000000E+00       2551.55       8031.70  0.000000E+00       12762.0       49.6914       10821.2 -1.000000E+10 -1.000000E+10 -1.000000E+10 -1.000000E+10 -1.000000E+10  0.000000E+00  0.000000E+00  0.000000E+00  0.000000E+00  0.000000E+00  0.000000E+00  0.000000E+00  0.000000E+00  0.000000E+00  0.000000E+00  0.000000E+00  0.000000E+00       Funtag_D

   MaxLik Scaling Cycle 0105

   Wilson plot

   Statistics in resolution bins after refinement cycle 0003
```


|  |  |
| --- | --- |
|  | - Rxpct factors plot- Rxpct factors table- <Log-Lik Gain>- Ln Sigma A- Correlation Coefficients |


```
   Iters     Tot_Funct    Xray_Funct    Geom_Funct          Grms     rmsD2init     maxD2init             R         Rfree           LLG       LLGfree     rms(bond)    rms(angle)
   Iters             K   B_IMPF_FRAG        K_MISS   B_TEMP_MISS   K_IMPF_MISS   B_IMPF_MISS        K_SOLV   B_TEMP_SOLV   K_IMPF_SOLV   B_IMPF_SOLV        B_TEMP     B_TEMP_11     B_TEMP_12     B_TEMP_22     B_TEMP_13     B_TEMP_23     B_TEMP_33      K_WILSON      B_WILSON      FGCYCLES
   Iters          BOND         ANGLE      IMPROPER       TORSION    SINTORSION      TRIGONAL        PLANAR  BCORRELATION       CONTACT     IDEALDIST        CHIRAL    SIMILARITY       OCCSSUM  UTILDISTANCE     UTILANGLE   UTILTORSION        RHOFIT        ADP-01        ADP-02        ADP-03        ADP-04        ADP-05        ADP-06        ADP-07        ADP-08        ADP-09        ADP-10      RHOFITPC            QM
     100  4.668729E+05  4.228507E+05       44022.2       6.35663     0.0968266       1.34280      0.187956      0.240397    0.00910722   -0.00827694    0.00801331       1.00169       Opttag_D
     100       83.4025       1.28304       1.00000  0.000000E+00       1.00000  0.000000E+00      0.941908       88.1133       1.00000       39.8690  0.000000E+00       9.31987  0.000000E+00       9.31987  0.000000E+00  0.000000E+00      -18.6397       83.8070       4.12606        1       Scatag_D
     100       1809.74       5478.49  0.000000E+00  0.000000E+00       2519.37  0.000000E+00       2550.81       8028.31  0.000000E+00       12763.4       49.7283       10822.3 -1.000000E+10 -1.000000E+10 -1.000000E+10 -1.000000E+10 -1.000000E+10  0.000000E+00  0.000000E+00  0.000000E+00  0.000000E+00  0.000000E+00  0.000000E+00  0.000000E+00  0.000000E+00  0.000000E+00  0.000000E+00  0.000000E+00  0.000000E+00       Funtag_D
   Geometry Restraint Deviations SCREEN Information

 
 No -bcorrel_zeroocchydrogens_factor flag found - keeping default: will set B for OCC=0 hydrogens to    1.200 times heavy-atom B


 TLS information. Total number of groups:     2


 Group   Natoms    FixRB   FixTLS   Group Tag    Group Spec
     1     2781      Y        Y     tls1    { A|* }
     2     2770      Y        Y     tls2    { B|* }
 
NOTE BUSTER_TLS_SET tls1 { A|* }
NOTE BUSTER_TLS_T   tls1     -9.27022E-02  3.86691E-02  3.74474E-02 -4.12978E-02  3.98790E-02 -2.98646E-02
NOTE BUSTER_TLS_L   tls1      1.77308E+00  1.37785E+00  1.02337E+00 -5.24463E-01 -1.21175E+00  5.33191E-01
NOTE BUSTER_TLS_S   tls1      9.10334E-02 -3.37935E-01  3.79187E-01 -3.08938E-01  1.66879E-01 -1.72939E-01 -3.02906E-02 -2.18204E-01
NOTE BUSTER_TLS_O   tls1     -2.30832E+01 -8.41146E+01  1.12764E+01
NOTE BUSTER_TLS_FIX tls1 ALL
 
NOTE BUSTER_TLS_SET tls2 { B|* }
NOTE BUSTER_TLS_T   tls2     -1.45544E-01 -8.72395E-02  1.24440E-01  1.38485E-02  3.79911E-02 -6.45887E-03
NOTE BUSTER_TLS_L   tls2      1.08099E+00  8.79887E-01  6.84078E-01  5.26234E-01 -7.68579E-01 -5.77148E-01
NOTE BUSTER_TLS_S   tls2     -5.61912E-02  1.02074E-01 -8.18820E-02 -1.38494E-02  1.32376E-01  4.36276E-02 -1.03695E-01  9.56923E-02
NOTE BUSTER_TLS_O   tls2      2.22817E+01 -7.23258E+01  3.51644E+00
NOTE BUSTER_TLS_FIX tls2 ALL

 For final position, Geometry function summary
     term                           number     weight       rms        GooF      function     fn/numb
     ================================================================================================
     BOND bond lengths (angs)       5665       2.000      0.0080       0.400      1809.74       0.319
     ANGL bond angles (degs)        7687       2.000        1.00       0.597      5478.49       0.713
     TORS split-harmonic tors       1275       0.000       18.24       1.147         0.00       0.000
     SINTOR sinusoidal tors         1275       2.000       18.24                  2519.37       1.976
     PLAN general plane (angs)       967       5.000      0.0145       0.726      2550.81       2.638
     BCORrelations (angs^2)         5665      10.000       1.055       0.376      8028.31       1.417
     CONT (bad contacts)               0       5.000                                 0.00
     IDEAL(ideal-dist contact)      5709       4.000                             12763.41
     CHIRAL (gelly semiharmon)       740       5.000                                49.73
     SIM similarity restraint for NCS and/or TARGET                              10822.33
     OCCSUM restraint on sum of occupanies                                           0.00       as none defined
     DISTAN utility restraints interatomic distance                                  0.00       as none defined
     UTILANGLE utility angle restraints                                              0.00       as none defined
     UTILTOR utility restraints on torsion angles                                    0.00       as none defined
     Peptide omega torsion angles                           2.92 degs - monitor only

 Weighted rms      0.0080         1.002  (temporary rms bond and angle for BUSTER to pick up!)
 Weighted intra-residual rms(bond) for StdProtein (excluding H)      0.0078
 Weighted intra-residual rms(bond) for Buffer     (excluding H)      0.0033
 Outputing geometry progress graph in plotmtv format to file:Geom_Res.mtv

 ############### Running final geometry/screen analysis (screen_final.txt)

 s/r gelly normal termination
```

Statistics and scale factors vs. cycle number
```

|  |  |  |
| --- | --- | --- |
|  | - Rxpct factors- <Log-Lik Gain>- GEOM Residuals- Ln Sigma_A_ | - Scale Factors- B Factors- Imperfection Factors |

```
   Final Geometry Information


---


### 2. Final results

Statistics and scale factors vs. cycle number
```

|  |  |  |
| --- | --- | --- |
|  | - Rxpct factors- <Log-Lik Gain>- GEOM Residuals- Ln Sigma_A_ | - Scale Factors- B Factors- Imperfection Factors |

```
   Average Figure of Merit
   Structure Factor Amplitudes


 # normal termination

  Program stopped.
```

closesok message: time spent in tree = 1760.57 (user) 31.97 (sys)

---

# Normal termination

```
 Resources usage summary:
     Time spent in client ...................  1760.58 (user)       31.99 (system)
     Time spent in scripts (    27 calls) ...     2.08 (user)        0.16 (system)
     Time spent in server ...................     0.00 (user)        0.01 (system)

     Max resident memory used (kb) .......... 379464
     Number of page faults .................. 0 (major)    690642 (minor)
     Number of context switches ............. 8272 (niced)    6826 (other)
```
